# Novel Pt@PCN-Cu-induced cuproptosis amplifies αPD-L1 immunotherapy in pancreatic ductal adenocarcinoma through mitochondrial HK2-mediated PD-L1 upregulation

**DOI:** 10.1186/s13046-025-03409-4

**Published:** 2025-05-17

**Authors:** Pengyu Wang, Weihua Guo, Shuyue Liu, Shouyi Li, Jiaqi Li, Bowen Ding, Fengyi Yin, Yang Yang, Xingjiang Li, Pei Cao, Chaozhe Ma, Wanying Zhang, Yidan Song, Yating Geng, Lantao Liu, Jing Hu, Jihui Hao, Yukuan Feng

**Affiliations:** 1 Pancreas Center, State Key Laboratory of Druggability Evaluation and Systematic Translational Medicine, Tianjin Key Laboratory of Digestive Cancer, Tianjin’s Clinical Research Center for Cancer, Tianjin Medical University Cancer Institute and Hospital, National Clinical Research Center for Cancer, Tianjin, 300060 China; 2 School of Basic Medicine, Mudanjiang Medical University, Mudanjiang, 157011 China; 3 School of Basic Medicine, Tianjin Medical University, Tianjin, 300070 China

**Keywords:** Cuproptosis, HK2, PD-L1, Tumor microenvironment, Immunotherapy, Nanozyme

## Abstract

**Background:**

Copper accumulation triggers mitochondrial-driven cell death, known as cuproptosis, offering a promising mechanism for targeted cancer therapy. Recent studies have highlighted the critical role of intratumoral copper levels in regulating the expression of programmed cell death ligand-1 (PD-L1), suggesting that copper-induced cuproptosis not only enhances cancer cell death but may also amplify the effects of anti-PD-L1 antibodies (αPD-L1). However, in tumors where monotherapy with αPD-L1 shows limited efficacy, particularly in pancreatic ductal adenocarcinoma (PDAC), the role of copper-induced cuproptosis in enhancing αPD-L1 treatment efficacy and its underlying mechanisms remain unclear. Meanwhile, inadequate tumor drug accumulation and glycolysis significantly restrict the efficacy of cuproptosis. To address these challenges, we have synthesized a novel nanozyme, Pt@PCN-Cu, designed to stabilize intracellular copper accumulation and effectively induce cuproptosis. Additionally, we aim to determine whether this strong induction of cuproptosis can synergize with αPD-L1 to enhance cancer therapy, ultimately paving the way for novel strategies to improve PDAC treatment.

**Methods:**

Pt@PCN-Cu was synthesized via a one-pot method, and its therapeutic potential was assessed in combination with αPD-L1 for the treatment of PDAC. Initially, the material’s properties were characterized, and its efficient cellular uptake was confirmed. Anti-tumor efficacy was evaluated by inducing cuproptosis in PDAC cell lines and xenograft models. RNA sequencing (RNA-seq) was utilized to identify key regulators involved in the modulation of PD-L1 expression by cuproptosis. Lastly, the therapeutic efficacy of Pt@PCN-Cu combined with αPD-L1 was evaluated in vivo, focusing on tumor growth inhibition and immune modulation within the tumor microenvironment (TME).

**Results:**

Pt@PCN-Cu demonstrates excellent physicochemical properties and remarkable cascade catalytic activity, providing a solid foundation for further in vitro and in vivo studies. In vitro, Pt@PCN-Cu efficiently transports copper and induces cuproptosis primarily through mitochondrial dysfunction. Mechanistic studies show that Pt@PCN-Cu triggers the dissociation of hexokinase 2 (HK2) from mitochondria, leading to a reduction in HK2 activity. This decline in HK2 activity impairs glycolysis, a metabolic pathway essential for tumor energy metabolism, which in turn results in elevated PD-L1 levels. In vivo, Pt@PCN-Cu demonstrates excellent safety and accumulates at the tumor site in a subcutaneous PDAC mouse model, inducing cuproptosis. Moreover, the combination of Pt@PCN-Cu with αPD-L1 further enhanced its therapeutic efficacy and effectively reprogrammed the immunosuppressive TME.

**Conclusion:**

This study presents strong evidence confirming the safety and therapeutic potential of Pt@PCN-Cu in PDAC treatment. Importantly, Pt@PCN-Cu not only induces cuproptosis but also significantly enhances antitumor efficacy in combination with αPD-L1 by regulating PD-L1 expression through HK2 modulation. These findings underscore a more effective and innovative approach for treating PDAC.

**Supplementary Information:**

The online version contains supplementary material available at 10.1186/s13046-025-03409-4.

## Introduction

Pancreatic ductal adenocarcinoma (PDAC), the most common form of pancreatic cancer, is associated with a poor prognosis, with a 5-year survival rate of only 10%, representing a significant threat to patient survival [1, 2]. Approximately 80% of patients are diagnosed at advanced or metastatic stages, making them ineligible for surgical resection [3]. For these individuals, chemotherapy remains the primary treatment, though its efficacy is substantially compromised by both primary and acquired resistance, posing a major challenge to clinical management [4, 5]. Immune checkpoint inhibitors (ICIs), such as anti-programmed cell death ligand-1 (αPD-L1) antibodies, have shown considerable efficacy in treating various solid tumors, leading to improved survival rates [6]. However, PDAC continues to exhibit limited response to immunotherapy. This is partly due to tumor-associated macrophages (TAMs), regulatory T cells (Tregs) and myeloid-derived suppressor cells (MDSCs), which contribute to CD8⁺ cytotoxic T lymphocyte (CD8⁺ T cell) dysfunction, thereby creating a highly immunosuppressive tumor microenvironment (TME) that facilitates immune evasion [7]. Additionally, low PD-L1 expression in PDAC further reduces the effectiveness of αPD-L1 therapy [8]. Therefore, it is critical to develop a treatment strategy that not only induces tumor cell death but also reverses the immunosuppressive TME to enable more effective anti-tumor responses.

Copper-induced cell death, termed cuproptosis, is regulated by enzymes such as lipoyl synthase (LIAS) and ferredoxin 1 (FDX1), which govern the lipoylation of mitochondrial enzymes. Copper binding to dihydrolipoamide S-acetyltransferase (DLAT) disrupts the normal lipidation of these enzymes, leading to mitochondrial dysfunction. This disturbance destabilizes Fe-S cluster proteins, further impairing mitochondrial function and cellular metabolism, ultimately resulting in toxicity and cell death [9]. Therefore, cuproptosis is characterized by copper-induced disruption of mitochondrial lipidation, destabilizing crucial enzymes and metabolic pathways, which in turn causes mitochondrial damage and cell death [10]. The dysregulation of copper metabolism in tumors highlights cuproptosis as a promising therapeutic target [11]. Emerging evidence suggests that cuproptosis may also influence PD-L1 expression in cancer cells, although the underlying mechanisms remain unclear [12]. PD-L1 expression is a critical biomarker for ICIs, and PD-L1 expression levels are commonly used to guide αPD-L1 therapy and predict immunotherapy outcomes [13]. Although the direct connection between cuproptosis and PD-L1 regulation remains incompletely understood, copper accumulation may indirectly influence PD-L1 expression levels, thereby modulating tumor-immune interactions [14]. This potential interaction suggests that cuproptosis may enhance the therapeutic effect of αPD-L1 by modulating PD-L1 expression, thereby improving the TME and promoting the functional recovery of CD8^+^ T cells. However, further investigation is required to validate this hypothesis and explore the underlying mechanisms.

Cuproptosis is associated with mitochondrial damage and disrupted metabolic processes, with glycolysis potentially playing a role in this mechanism. Studies have shown that upregulation of hexokinase 2 (HK2) correlates with enhanced glycolysis and tumorigenesis, while HK2 inactivation reduces glycolysis and suppresses tumor growth and metastasis [15, 16]. Additionally, the inactivation of HK2 redirects glycolytic flux toward mitochondrial metabolism, which is considered vital for increasing cellular sensitivity to cuproptosis (cells that rely more on mitochondrial respiration are nearly 1000-fold more sensitive to Cu⁺ than cells that primarily rely on glycolysis) [17, 18]. While these studies suggest a possible link between cuproptosis and HK2, the specific mechanisms by which cuproptosis regulates HK2 and the factors involved remain to be clarified.

Targeting cuproptosis as a therapeutic strategy for cancer holds significant promise but is accompanied by notable challenges and limitations. The primary challenges lies in selectively delivering therapeutic agents into tumor cells while minimizing systemic exposure and associated toxicity. To address this, advanced drug delivery strategies require further optimization to enhance tumor specificity and minimize off-target effects. These approaches aim to enhance therapeutic efficacy while minimizing adverse outcomes, underscoring the need for innovative solutions to overcome these challenges. Currently, nanozymes (enzyme-like nanomaterials) have garnered special attention due to their low cost, high stability, multifunctionality and ease of large-scale production [19, 20]. Nanozymes could achieve multiple therapeutic effects and enhance the efficiency of tumor treatment by combining multiple reaction steps through their catalytic properties [21]. However, due to the requirement for mutually incompatible reaction conditions, there are currently only a limited number of reported nanozyme cascade reaction systems.

In this study, we developed a cascade nanozyme (Pt@PCN-Cu) that mimics the functions of catalase and copper ionophores to promote PD-L1 expression via an HK2-dependent mechanism. Moreover, Pt@PCN-Cu, in combination with αPD-L1, reprograms the immunosuppressive TME, thereby stimulating robust anti-tumor immune responses. In conclusion, Pt@PCN-Cu provides a promising strategy for inducing cuproptosis, which, when paired with αPD-L1 therapy, significantly improves anti-PDAC efficacy, with strong potential for clinical application.

## Materials and methods

### Chemical materials

ZrOCl_2_·8H_2_O (13520-92-8), benzoic acid (65-85-0), chloroplatinic acid (H_2_PtCl_6_·6H_2_O, 18497-13-7), agarose gel (9012-36-6) and N, N-dimethylformamide (DMF, 68-12-2) were bought from Aladdin Biochemical technology (Shanghai, China). All chemical reagents were of analytical grade and were used without further purification. Deionized water was used to prepare all aqueous solutions.

### Synthesis of Pt@PCN-Cu and Pt@PCN-Cu@Cy5.5

The Pt@PCN-Cu nanoparticles (NPs) were synthesized following a previously reported method with minor modifications [22, 23]. Briefly, TCPP-Cu (22 mg), ZrOCl₂·8 H₂O (60 mg) and benzoic acid (580 mg) were dissolved in 20 mL of DMF and stirred magnetically at 90 °C for 5 h at 300 rpm. After cooling to room temperature, the PCN-Cu NPs were collected by centrifugation at 12,000 rpm for 20 min and washed multiple times with DMF. The PCN-Cu NPs were redispersed in DMF for storage.

For the synthesis of Pt NPs, PVP (266 mg, Mw = 24000) was dissolved in 90 mL of methanol, followed by the addition of 10 mL of 50 mM H_2_PtCl_6_. The mixture was refluxed under air for 3 h. Methanol was removed using a rotary evaporator, and the Pt NPs were precipitated by acetone for 3 h, then collected by centrifugation at 6000 rpm for 5 min. The obtained Pt NPs were further purified using chloroform and hexane, then redispersed in DMF for storage.

The Pt@PCN-Cu NPs were synthesized by dissolving TCPP-Cu, ZrOCl_2_·8H_2_O, and benzoic acid in DMF, followed by the addition of as-synthesized PVP-Pt NPs (10 mM). The mixture was stirred at 300 rpm at 90 °C for 5 h. The resulting Pt@PCN-Cu NPs were collected by centrifugation, washed multiple times with DMF, and redispersed in DMF for storage. Pt@PCN-Cu@Cy5.5 NPs were synthesized using a similar method, with the addition of a small amount of Cy5.5 to the DMF. The mixture was then centrifuged at 12,000 rpm for 15 min and washed five times with ultrapure water until the supernatant was free of fluorescent dye.

### Material characterization

The morphology of PCN-Cu, Pt@PCN-Cu and Pt NPs was analyzed using transmission electron microscopy (TEM). Dynamic light scattering (DLS) and surface zeta potential measurements were conducted using a Malvern Zetasizer Nano Analyzer. X-ray photoelectron spectroscopy (XPS) was performed with a Thermo Scientific K-Alpha instrument. The UV-vis absorption spectrum was measured using a UV-vis spectrometer.

### GSH depletion

To evaluate GSH depletion, Pt@PCN-Cu at various concentrations (0, 2, 5, 10, and 20 nM) was incubated with a GSH solution (10 mmol/L) at 37℃ for 20 min. Following incubation, 5,5’-Dithio-bis-(2-nitrobenzoic acid) (DTNB) solution (3 mg/mL) was added to each mixture and stirred for 5 min. The absorbance at 412 nm was then measured using a multimode plate reader.

### Copper ion generation

Pt@PCN-Cu (20 nM) was incubated with varying concentrations of GSH (0, 2.5, 5, 10 and 20 mmol/L) for 1 h at 37 °C. The fluorescence intensity at 608 nm was measured using a microplate reader.

### Cell lines

PANC-1, KPC, HEK293T, hTERT-HPNE, AML12, and dendritic cells were cultured in Dulbecco’s Modified Eagle’s Medium (DMEM), while SW1990 and RAW 264.7 cells were maintained in Roswell Park Memorial Institute (RPMI) 1640 medium. CFPAC-1 cells were cultured in Iscove’s Modified Dulbecco’s Medium (IMDM). All media were supplemented with 10% fetal bovine serum (FBS) and 1% penicillin/streptomycin (P/S), and cells were incubated at 37 °C in a 5% CO₂ incubator. Transfection was performed using Lipofectamine^®^ 2000 reagent (11668027, Invitrogen, USA) according to the manufacturer’s protocol. For glucose-6-phosphate treatment, G-6-P solution (1 M) was mixed with 5 mL of Lipofectamine^®^ 2000 in OPTI-MEM for 30 min at room temperature. The resulting mixture was then added to the culture medium in a 6-well plate.

### Animals

Male C57BL/6 mice (4 weeks old) were purchased from SPF (Beijing) biotechnology Co. Ltd. and raised in specific pathogen-free (SPF) animal facilities. All animal experiments were conducted in accordance with guidelines approved by the Committee of Animal Experimentation and the Ethics Committee of Tianjin Medical University Cancer Institute and Hospital (Approval Number: NSFC-AE-2024344).

### In vitro cellular uptake of Pt@PCN-Cu

A coverslip was placed at the bottom of each well in a 12-well plate. PANC-1 cells (1 × 10^5^) suspended in 1 mL of medium were seeded into each well and incubated overnight at 37 °C. The cells were then treated with Pt@PCN-Cu for 1 h, 4 h, and 8 h, respectively. Following treatment, the culture medium was removed, and the cells were washed three times with PBS before being incubated in fresh medium. The coverslip was mounted onto a microscope slide, and the cell nuclei were stained with DAPI, while F-actin filaments were stained with Phalloidin. Finally, images were captured using confocal laser scanning microscopy (CLSM).

### Copper cellular uptake determined by atomic absorption spectroscopy (AAS)

PANC-1 cells were seeded into 6-well plates at a density of 1 × 10^6^ cells per well and incubated overnight at 37 °C. The cells were then treated with PBS, ES (HY-12040, MedChemExpress, Shanghai, China), CuCl_2_ (7447-39-4, Sigma-Aldrich, Beijing, China) + ES or Pt@PCN-Cu. After 2 h incubation at 37 °C, the cells were washed three times with PBS and acidified with nitric acid. The copper content in the cell lysates was subsequently quantified using AAS.

### Intracellular copper content assay

PANC-1 cells (1 × 10^6^) were seeded in 6-well plates and incubated overnight. Pt@PCN-Cu was incubated with PANC-1 cells for 8 h, after which the cells were washed with PBS three times. Then, the cell sample was digested with 2% nitric acid at 60 °C for 2 h. The intracellular copper concentration in the cell lysis solution was determined via inductively coupled plasma-mass spectrometry (ICP-MS) analysis.

### Cell viability assays

Cell viability was assessed using the MTT assay (C0009S, Beyotime, Shanghai, China). PANC-1, SW1990 and CFPAC-1 cells were seeded in 96-well plates at a density of 5000 cells per well and incubated for 24 h to allow attachment. The cells were then treated with various concentrations of ES, CuCl_2_ + ES and Pt@PCN-Cu (the concentrations of ES and Pt@PCN-Cu were varied at 0.1, 0.25, 0.5, 1, 2.5, 10, 20, 50 and 100 nM) for 24 h. After treatment, 10 µL of MTT solution was added to each well, and the cells were incubated for 4 h at 37 °C. The absorbance was measured at 570 nm using a microplate reader.

PANC-1, AML12, hTERT-HPNE, RAW 264.7 and dendritic cells were seeded in 96-well plates at a density of 5000 cells per well and incubated for 24 h to allow attachment. The cells were then treated with various concentrations of Pt@PCN-Cu for 24 h. After treatment, 10 µL of MTT solution was added to each well, and the cells were incubated for 4 h at 37 °C. The absorbance was measured at 570 nm using a microplate reader.

### Live-Dead cell staining

Live/dead cell viability was assessed in PANC-1 cells using a Calcein/PI cell viability/cytotoxicity assay kit (C2015M, Beyotime, Shanghai, China). PANC-1 cells were seeded in confocal dishes and allowed to adhere for 24 h. Subsequently, the cells were treated with ES (50 nM), CuCl_2_ + ES (5 µM + 50 nM) and Pt@PCN-Cu (20 nM) for 24 h. After treatment, the cells were washed with PBS and co-stained with Calcein-AM and PI. Fluorescence images were captured using CLSM, with distinct fluorescence channels for green and red signals.

### Apoptosis analysis

Cellular apoptosis was evaluated using an APC-Annexin V and PI apoptosis kit (A6030, US EVERBRIGHT, Suzhou, China). Briefly, PANC-1 cells were seeded in 6-well plates and treated with PBS, ES (50 nM), CuCl_2_ + ES (5 µM + 50 nM) or Pt@PCN-Cu (20 nM) for 24 h. After treatment, cells were harvested and stained following the manufacturer’s protocol. Apoptosis was assessed by flow cytometry using a flow cytometer. Data were analyzed with FlowJo software, and apoptotic cell populations were quantified based on Annexin V and PI staining.

### Colony formation assay

PANC-1 cells were seeded in 6-well plates at a density of 2000 cells per well and incubated for 24 h. The cells were then treated with PBS, ES (50 nM), CuCl_2_ + ES (5 µM + 50 nM) or Pt@PCN-Cu (20 nM) for 24 h. Following treatment, the medium was replaced, and cells were cultured for an additional 7 days. After treatment, cells were fixed with 4% paraformaldehyde (BL539A, Biosharp, Beijing, China) for 15 min and stained with 0.2% crystal violet (C0121, Beyotime, Shanghai, China) for 10 min. The colonies were rinsed with ultrapure water to eliminate any residual stain, photographed using a digital camera, and subsequently quantified with Image J software.

### Enhanced mitochondrial membrane potential assay kit with JC-1 staining

Mitochondrial membrane potential was assessed using the JC-1 staining kit (C2003S, Beyotime, Shanghai, China), following the manufacturer’s protocol. After treatment, cells were incubated with the JC-1 staining solution for 20 min, and excess stain was removed by washing with the provided buffer. Fluorescent images were captured using an inverted fluorescence microscope. Red and green fluorescence intensities were evaluated to assess changes in mitochondrial membrane potential.

### Intracellular reactive oxygen species (ROS) detection

Intracellular ROS levels were measured using ROS assay kit (S0033S, Beyotime, Shanghai, China). Briefly, a cover slip was placed in the bottom of each well of a 12-well plate, and PANC-1 cells (1 × 10^5^) were seeded in 1 mL of complete medium and incubated at 37 °C for 16 h. Following this, the cells were treated with PBS, ES (50 nM), CuCl_2_ + ES (5 µM + 50 nM) or Pt@PCN-Cu (20 nM) for 8 h. After treatment, the culture medium was replaced with serum-free medium, and cells were incubated with the ROS indicator DCFH-DA (10 µM) for 30 min. The cover slips were then transferred onto microscope slides and the fluorescent images were acquired using CLSM.

### Observation of mitochondrial morphology

PANC-1 cells were seeded into 6-well plates at a density of 1 × 10^6^ cells per well and cultured for 12 h. The cells were then treated with Pt@PCN-Cu (20 nM) for 24 h. After treatment, the cells were harvested, fixed with 2.5% glutaraldehyde (DF0166, Leagene, Beijing, China), and processed for TEM analysis.

### LDH release assay

PANC-1 cells (1 × 10^4^ cells) were seeded into 96-well plates and incubated overnight. The following day, the cells were treated with PBS, ES (50 nM), CuCl₂+ES (5 µM + 50 nM) or Pt@PCN-Cu (20 nM) for 24 h. Subsequently, extracellular LDH levels were measured using the LDH cytotoxicity assay kit (C0016, Beyotime, Shanghai, China), following the manufacturer’s instructions.

### ATP assays

PANC-1 cells (1 × 10^4^) were seeded into 96-well plates and incubated overnight. The following day, the cells were treated with PBS, ES (50 nM), CuCl_2_ + ES (5 µM + 50 nM) or Pt@PCN-Cu (20 nM) for 24 h. Subsequently, intracellular ATP concentrations were measured using the ATP assay kit (S0027, Beyotime, Shanghai, China) in accordance with the manufacturer’s instructions.

### Intracellular GSH assay

Intracellular GSH levels were assessed using the GSH assay kit (S0053, Beyotime, Shanghai, China). PANC-1 cells were seeded into 6-well plates at a density of 4 × 10^5^ cells per well and cultured for 24 h. The cells were then treated with PBS, ES (50 nM), CuCl_2_ + ES (5 µM + 50 nM) or Pt@PCN-Cu (20 nM) for 24 h. After treatment, intracellular GSH levels were measured using the GSH assay kit according to the manufacturer’s instructions.

### Glucose uptake measurement

Glucose uptake was measured using the glucose uptake assay kit (ab136956, Abcam, U.K.) according to the manufacturer’s protocol. Initially, PANC-1 cells were treated with PBS, ES (50 nM), CuCl_2_ + ES (5 µM + 50 nM) or Pt@PCN-Cu (20 nM) for 24 h. The cells were then incubated for 20 min in Krebs-Ringer-Phosphate-Hepes (KRPH) buffer (K4002, Sigma-Aldrich, Beijing, China) containing 2% bovine serum albumin. After this, 2-deoxy-D-glucose (2-DG) was added to the medium for an additional 20-minute incubation. Following treatment, the cells were lysed using the provided extraction buffer, frozen at -80 °C for 15 min, and then heated at 85 °C for 40 min. After cooling, neutralization buffer was added to the lysates, which were then centrifuged at 12,000 rpm for 5 min to collect the supernatant. Glucose uptake was quantified by measuring the absorbance at 412 nm using a microplate reader.

### Lactate assay

Lactate levels were measured using the lactate assay kit (ab65331, Abcam, U.K.) according to the manufacturer’s instructions. Briefly, PANC-1 cells were treated with PBS, ES (50 nM), CuCl_2_ + ES (5 µM + 50 nM) or Pt@PCN-Cu (20 nM) for 24 h. Following treatment, the cells were processed in ice-cold lactate assay buffer. The samples were then centrifuged to collect the supernatant, which was deproteinized using the deproteinizing sample preparation kit-TCA (ab204708, Abcam, U.K.). After adding the reaction mix, the samples were incubated at room temperature for 30 min.

### Knockdown of HK2 expression

Lentiviral particles were generated in HEK293T cells and used to infect PANC-1 cells to suppress HK2 gene expression. The shRNA targeting sequence for HK2 was 5′-GGATGTGTGTGAACATGGAAT-3′. All procedures were performed following standard protocols.

### Western blot

PANC-1 cells were divided into four treatment groups: (1) PBS, (2) ES, (3) CuCl_2_ + ES, and (4) Pt@PCN-Cu. After 24 h of incubation, cell lysates were prepared by adding cell lysis buffer and extracting proteins via centrifugation at 12,000 rpm for 10 min. The protein concentration was determined using a BCA protein assay kit (P0012, Beyotime, Shanghai, China). Equal amounts of protein were separated by sodium dodecyl sulfate-polyacrylamide gel electrophoresis (SDS-PAGE), followed by transfer to a PVDF membrane using a gel electrophoretic apparatus. After blocking with 5% milk for 1 h, the membranes were incubated overnight at 4 °C with primary antibodies against DLAT (13426-1-AP, Proteintech, Wuhan, China), LIAS (11577-1-AP, Proteintech, Wuhan, China), FDX1 (12592-1-AP, Proteintech, Wuhan, China), PD-L1 (13684 S, Cell Signaling Technology, U.S.A), HK2 (ab209847, Abcam, U.K.), VDAC1 (66345-1, Proteintech, Wuhan, China), COX IV (4844 S, Cell Signaling Technology, USA), and β-actin (81115-1-RR, Proteintech, Wuhan, China). After washing three times with TBST, the membranes were incubated with secondary antibodies for 1 h at room temperature. Western blot signals were detected using a Gel Imaging System after incubation with ECL chemiluminescent reagent.

### RNA extraction and quantitative Real-Time PCR (qRT-PCR)

Total RNA was isolated from cells using TRIzol reagent (15596018CN, Invitrogen, USA), according to the manufacturer’s instructions. The quality and concentration of the extracted RNA were assessed using a NanoDrop spectrophotometer. Subsequently, 1 µg of total RNA was reverse transcribed into first-strand cDNA using the revertAid first strand cDNA synthesis kit (K1621, Thermo Fisher Scientific, USA). The qRT-PCR was performed using SYBR Premix Ex Taq™ II (RR390A, TaKaRa, Japan), following the manufacturer’s guidelines. The expression levels of the target genes were normalized to β-actin, and relative gene expression was calculated using the 2-ΔΔCt method.

The following primers were used for qRT-PCR, PD-L1 forward:5’-CTGCACTTTTAGGAGATTAGATC-3’; reverse: 5’-CTACACCAAGGCATAATAAGATG-3’; β-actin forward: 5’-TGGCACCCAGCACAATGAA-3’; reverse: 5’-CTAAGTCATAGTCCGCCTAGAAGCA-3’.

### Subcellular fractionation

Mitochondrial and cytosolic fractions were isolated using the mitochondria/cytosol fractionation kit (ab65320, Abcam, U.K.) following the manufacturer’s instructions.

### Immunoprecipitation assay

An immunoprecipitation assay was conducted using protein extracts from PANC-1 cells. Briefly, the interaction between HK2 and VDAC1 was analyzed using the pierce™ crosslinking magnetic IP/Co-IP kit (88805, Thermo Fisher Scientific, USA) according to the manufacturer’s instructions.

### Hexokinase activity assay

Hexokinase activity was measured using the hexokinase activity assay kit (ab136957, Abcam, U.K.) according to the manufacturer’s instructions. Briefly, PANC-1 cell lysates were prepared and diluted in assay buffer. The reaction was initiated by adding a reaction mix containing glucose, ATP, G-6-P dehydrogenase, and NADP^+^, followed by incubation at room temperature for 30 min. The resulting colorimetric signal was measured at 450 nm using a microplate reader.

### Establishment of tumor model and therapeutic effect I

C57BL/6 mice were used to establish subcutaneous tumor models. Subcutaneous tumors were induced by injecting 1 × 10^6^ KPC tumor cells suspended in 100 µL of PBS into the forelimb axilla of each mouse. The mice were randomly assigned to four treatment groups: PBS, ES (5 mg/kg), CuCl_2_ + ES (1 mg/kg + 5 mg/kg) and Pt@PCN-Cu (5 mg/kg). The mice were intravenously administered their respective treatments on days 0, 3, 6, 9 and 12. Tumor volume was measured every three days throughout the study. Tumor volume was calculated using the formula: V=(x×y^2^)/2, where x and y represent the length and width of the tumor, respectively.

### In vivo biocompatibility evaluation

Mice were monitored for general health and weighed every four days throughout the study. Twelve days post-administration, the mice were euthanized, and blood samples were collected for serum biochemical analysis. Plasma was separated by centrifugation at 3000 rpm for 30 min. The levels of alanine aminotransferase (ALT), aspartate aminotransferase (AST), creatine kinase (CK), serum creatinine (Crea), direct bilirubin (DBIL), total bilirubin (TBIL) and urea were measured using standard biochemical assays. Major organs (heart, liver, spleen, lungs and kidneys) were dissected, fixed in 4% paraformaldehyde solution, and embedded in paraffin. Tissue sections were prepared and stained with hematoxylin and eosin (H&E, C0105S, Beyotime, Shanghai, China) to observe any pathological changes.

### In vivo biodistribution study

Tumor-bearing mice were intravenously injected with Cy7.5-labeled Pt@PCN-Cu (Pt@PCN-Cu@Cy7.5, 847180-48-7, MedChemExpress, Shanghai, China). In vivo biodistribution images were acquired at 2, 8 and 24 h post-injection. For the ex vivo biodistribution study, mice were euthanized 24 h after injection. The major organs, including the heart, liver, spleen, lungs, kidneys, intestine, and tumor, were harvested. Ex vivo imaging of these organs was performed using the In Vivo Imaging System (IVIS) to assess the distribution of Pt@PCN-Cu@Cy7.5. Additionally, Pt@PCN-Cu@Cy7.5 and Cu²⁺ in excised tumor tissues were analyzed using flow cytometry and ICP-MS, respectively.

### Histopathological analysis I

Solid tumors were excised from tumor-bearing mice for histological analysis. The excised tumors were fixed in 4% paraformaldehyde, embedded in paraffin, and sectioned for subsequent staining. H&E staining was performed for general histopathological examination. Ki67 (27309-1-AP, Proteintech, Wuhan, China) immunohistochemistry (IHC) staining was conducted to assess cell proliferation in tumor tissues.

Freshly dissected tumor tissues were fixed in 4% paraformaldehyde, dehydrated, and embedded in paraffin wax. Tissue sections were then prepared for further analysis. Following the manufacturer’s protocol, the sections were incubated overnight with primary antibodies against DLAT. After incubation with fluorescent secondary antibodies and DAPI, immunofluorescence images were captured using a CLSM.

### Establishment of tumor model and therapeutic effect II

C57BL/6 mice were used to establish subcutaneous tumor models by injecting 1 × 10^6^ KPC tumor cells (suspended in 100 µL PBS) into the flank region of each mouse. The mice were randomly assigned to four treatment groups: PBS, αPD-L1 (10 mg/kg, BE0101, BioXcell, USA), Pt@PCN-Cu (5 mg/kg) and Pt@PCN-Cu + αPD-L1. The treatment regimen was as follows: Pt@PCN-Cu was administered intravenously on days 0, 3, 6, 9 and 12, while αPD-L1 was administered intravenously on days 1, 4, 7, 10 and 13. Tumor volume was measured every three days throughout the study. Tumor volume was calculated using the formula: V=(x×y^2^)/2, where x and y represent the length and width of the tumor, respectively. For the survival studies, mice were euthanized when the tumor volume exceeded 1500 mm^3^. Survival data for each group were analyzed using the Kaplan-Meier method.

### Histopathological analysis II

Tumor specimens were harvested from tumor-bearing mice for histological analysis. The excised tumors were fixed in 4% paraformaldehyde at 4 °C for 24 h, followed by paraffin embedding. Tissue sections were prepared for subsequent staining. H&E staining was performed for general histopathological evaluation. IHC for Ki67 was carried out to assess cell proliferation.

### Flow cytometric analysis

For cell surface staining, cells were harvested and washed twice with PBS. Surface staining was performed by incubating the cells with antibodies, at the recommended dilution, in PBS on ice for 30 min. Following incubation, the cells were washed twice with PBS and fixed with 2% paraformaldehyde prior to flow cytometry analysis. For intracellular transcription factor Foxp3 staining, cells were first stained with surface markers, then fixed and permeabilized using the transcription factor buffer set (562574, BD, USA) according to the manufacturer’s instructions. The cells were subsequently incubated with antibodies for 30 min on ice. Flow cytometry was performed on a CytoFLEX LX (Beckman Coulter), and data were analyzed using FlowJo software. The following antibodies were used: APC/Fire^™^ 750 anti-mouse CD45 (30-F11), APC anti-mouse CD3 (17A2), PE/Dazzle^™^ 594 anti-mouse Ly-6 C (HK1.4), Brilliant Violet 421^™^ anti-mouse CD4 (RM4-5) and FITC anti-mouse CD8a (53 − 6.7) from BioLegend (USA). BUV661 Rat Anti-CD11b (M1/70), BB700 Rat Anti-Mouse Ly-6G (1A8) and the transcription factor buffer set from BD Biosciences (USA). PerCP-eFluor^™^ 710 FOXP3 monoclonal antibody (FJK-16s) from Thermo Fisher Scientific (USA). CD163 Monoclonal Antibody (TNKUPJ), Super Bright^™^ 600, eBioscience^™^ (TNKUPJ) from Thermo Fisher Scientific (USA). Brilliant Violet 421^™^ anti-mouse CD80 (16-10A1) from BioLegend (USA). Brilliant Violet 785^™^ anti-mouse F4/80 (BM8) from BioLegend (USA).

### Enzyme-Linked immunosorbent assay (ELISA)

After the respective treatments, blood samples were collected and allowed to clot at 4 °C for 30 min. The samples were then centrifuged at 3000 g for 15 min at 4 °C to separate the serum. Cytokine levels (IL-6, TNF-α and IFN-γ) were measured using commercially available ELISA kits(MM-1011M2, MM-0132M2 and MM-0182M2), following the instructions provided by Jiangsu Meimian Industrial Co., Ltd.

### RNA-Sequencing (RNA-seq)

Differentially expressed genes (DEGs) were identified using criteria of a fold change (FC) > 1.2 or < 0.83 and a false discovery rate (FDR) < 0.1. The raw data for all samples have been deposited in the NCBI Sequence Read Archive (SRA) under the accession number PRJNA1199319. The data can be accessed at the following link: https://www.ncbi.nlm.nih.gov/bioproject/PRJNA1199319.

### Statistical analysis

All statistical analyses were performed using GraphPad Prism 8. Data are presented as the mean ± standard deviation (SD) from at least three independent biological replicates, unless otherwise stated in the figure legend. For comparisons between two groups, t-test was used. For comparisons involving more than two groups, one-way analysis of variance (ANOVA) was applied. Statistical significance is indicated as follows: *P < 0.05, **P < 0.01 and ***P < 0.001.

## Results

### Preparation, characterization and cellular uptake of Pt@PCN-Cu

**Fig. 1:**
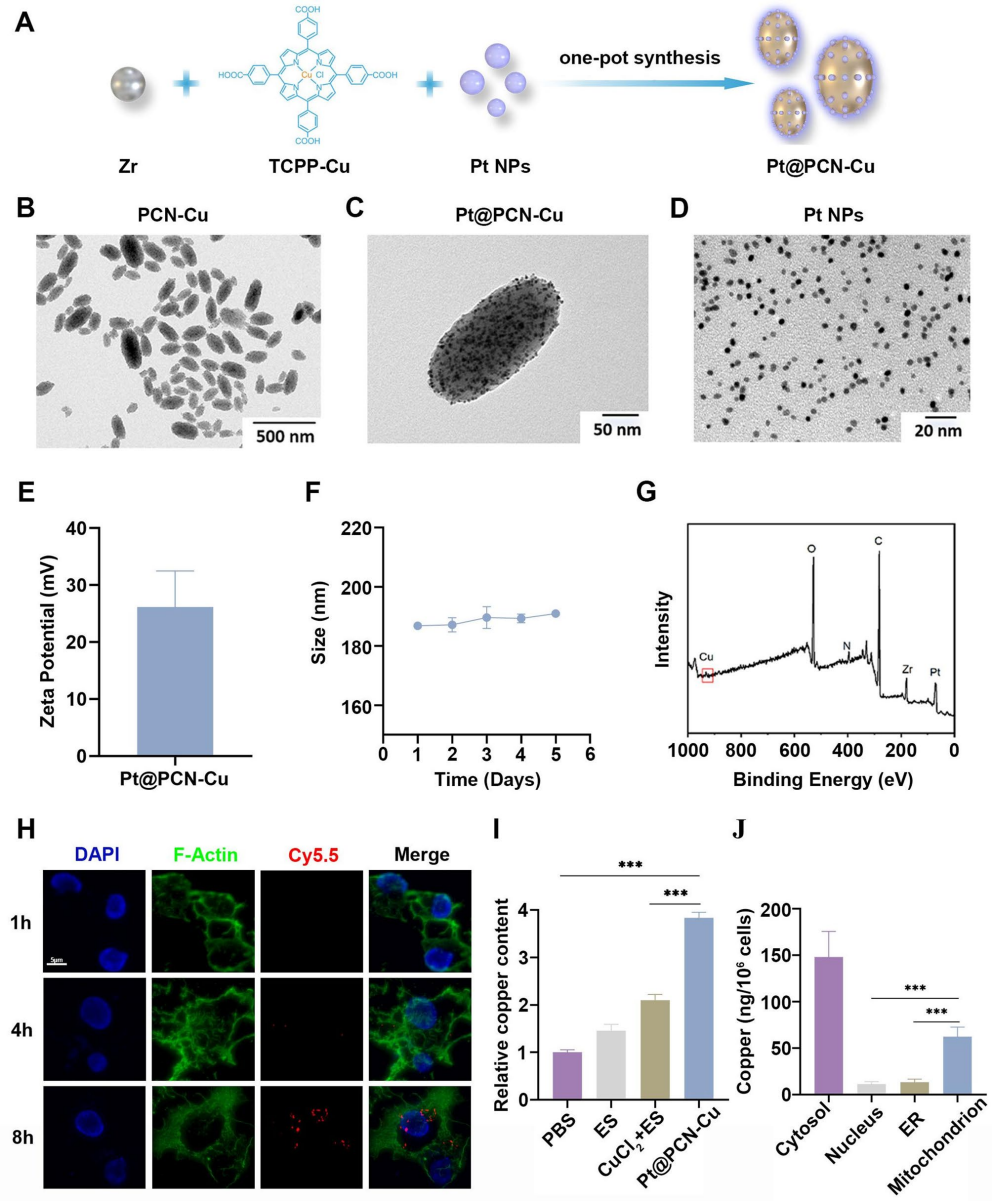
Synthesis, characterization and cellular uptake of Pt@PCN-Cu. (**A**) Schematic illustration of the synthesis procedure for Pt@PCN-Cu. (**B-D**) TEM images of PCN-Cu, Pt@PCN-Cu and Pt NPs. (**E**) Zeta potential of Pt@PCN-Cu. (**F**) Size changes of Pt@PCN-Cu in PBS after five days. (**G**) XPS analysis of Pt@PCN-Cu. (**H**) Representative CLSM images of PANC-1 cells incubated with Pt@PCN-Cu@Cy5.5 for 1, 4 and 8 h. Cell nuclei are stained with DAPI (blue) and F-actin filaments are stained with Phalloidin (green). Scale bar: 5 μm. (**I**) Intracellular copper uptake in PANC-1 cells at 2 h post-treatment with different formulations. (**J**) Subcellular distribution of copper in the cytosol, nucleus, ER and mitochondria of cells treated with Pt@PCN-Cu. Data are presented as mean ± SD. Statistical analysis was performed using one-way ANOVA, with ***P < 0.001

To enhance catalytic properties, a copper ionophore-like copper porphyrin moiety and catalase-mimicking platinum nanoparticles (Pt NPs) were integrated into the base metal-organic framework, PCN-Cu, resulting in the synthesis of Pt@PCN-Cu (Fig. 1A). The successful formation of PCN-Cu, Pt NPs and Pt@PCN-Cu was confirmed via transmission electron microscopy (TEM). As depicted in Fig. 1B and C, both PCN-Cu and Pt@PCN-Cu exhibited a uniform spindle-shaped structure with a diameter of approximately 180 nm. Meanwhile, Pt NPs displayed an average size of ~ 5 nm (Fig. 1D). Dynamic light scattering (DLS) analysis determined the hydrated particle size of Pt@PCN-Cu to be ~ 190 nm (Fig. S1A). The zeta potential of Pt@PCN-Cu was measured at 26.14 mV, with a polydispersity index (PDI) of 0.226, indicating excellent dispersity (Fig. 1E and Fig. S1B). The stability of Pt@PCN-Cu in PBS was evaluated using DLS. No significant changes in hydrodynamic particle size, zeta potential and PDI were observed after five days, suggesting good stability (Fig. 1F and Fig. S1C, D). The positively charged surface of NPs promote their uptake by immune cells, potentially resulting in cytotoxic effects [24]. As shown in Figure S2, Pt@PCN-Cu displayed little cytotoxic effect on dendritic cells (DCs) and RAW 264.7 cells compared with PANC-1 cells with the increase of the concentration. Furthermore, X-ray photoelectron spectroscopy (XPS) analysis confirmed the presence and valence states of Cu, C, N, Zr, and Pt in Pt@PCN-Cu (Fig. 1G). The XPS survey spectrum verified the successful incorporation of these elements into the Pt@PCN-Cu.

Extracellular Cu^2+^ ions are transported into the cell via copper ion transporters, or alternatively, extracellular transport enzymes facilitate the transfer of Cu^+^ ions [9]. Glutathione (GSH) undergoes oxidation upon interaction with Cu^2+^ ions, resulting in the formation of oxidized glutathione (GSSG) and Cu^+^. The accumulation of Cu^+^ ions is a key inducer of cuproptosis, making this oxidation process crucial for maintaining cellular redox balance and regulating copper ion metabolism. We evaluated the GSH-depleting ability of Pt@PCN-Cu using DTNB. The absorbance of TNB decreased significantly with increasing Pt@PCN-Cu concentrations (Fig. S3A). Subsequently, the GSH-triggered generation of Cu⁺ was confirmed using fluorescence spectroscopy, which revealed a notable increase in fluorescence intensity upon interaction with GSH, indicating the reduction of Cu^2+^ to Cu^+^ during GSH depletion (Fig. S3B). This suggests that Pt@PCN-Cu fulfills the essential prerequisites for triggering cuproptosis. To investigate the intracellular uptake of Pt@PCN-Cu, the particles were labeled with Cy5.5 dye (red), forming Pt@PCN-Cu@Cy5.5. As shown in Fig. 1H, the red fluorescence intensity progressively increased over time, demonstrating time-dependent cellular uptake of Pt@PCN-Cu.

Elesclomol (ES), a well-characterized copper ionophore, has been shown to effectively facilitate the transport of copper ions from extracellular sources into tumor cells. To assess the copper accumulation efficiency of various formulations, PANC-1 cells were subjected to three different treatments: ES, CuCl_2_ + ES and Pt@PCN-Cu. Intracellular copper content was subsequently quantified using atomic absorption spectroscopy (AAS) to compare cellular copper uptake across the different treatment groups. The results showed that cells treated with Pt@PCN-Cu exhibited copper content four times higher than those treated with PBS (Fig. 1I). Additionally, subcellular copper distribution analysis revealed that copper accumulation in mitochondria was significantly higher compared to the endoplasmic reticulum (ER) and nucleus (Fig. 1J). These findings demonstrate that Pt@PCN-Cu is effectively internalized by PANC-1 cells, where it promotes copper release in close proximity to the mitochondria.

### In vitro antitumor activity of Pt@PCN-Cu

**Fig. 2:**
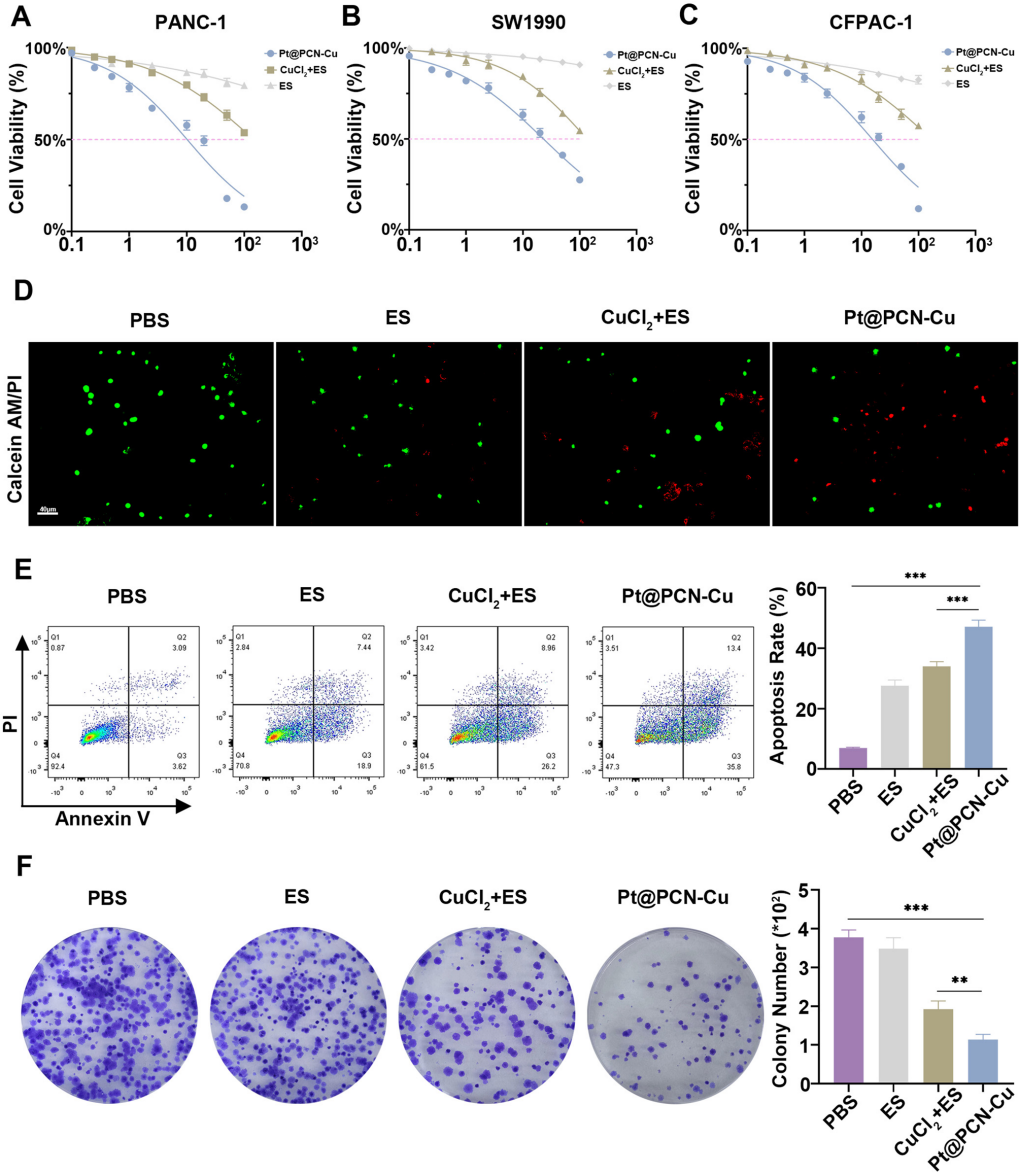
In vitro antitumor activity of Pt@PCN-Cu. (**A-C**) Cell viability of PANC-1, SW1990 and CFPAC-1 cells following various treatments, assessed using the MTT assay. (**D**) Representative CLSM images of PANC-1 cells after different treatments. Cells were stained with Calcein-AM and PI. Scale bar: 40 μm. (**E**) Flow cytometry (FCM) analysis showing the apoptotic rate of PANC-1 cells subjected to different treatments, quantified via APC-Annexin V/PI staining. (**F**) Representative images of colony formation assays in PANC-1 cells after treatment with different drugs. Data are presented as mean ± SD. Statistical analysis was performed using one-way ANOVA, with **P < 0.01 and ***P < 0.001

We next evaluated the in vitro cytotoxicity of Pt@PCN-Cu. Notably, an increase in intracellular copper concentration is directly associated with enhanced cytotoxicity of the drug [25]. In our study, the total intracellular copper content in the Pt@PCN-Cu-treated group was significantly elevated, reaching levels twice as high as those in the CuCl_2_ + ES-treated group (Fig. 1I). This finding indicates that Pt@PCN-Cu is more effective in inducing cell cuproptosis. Consistent with the aforementioned findings, Pt@PCN-Cu exhibited an exceptionally low IC50 value of 10.18 nM in PANC-1 cells, highlighting its potent cytotoxicity (Fig. 2A). A similar pattern was observed in SW1990 and CFPAC-1 cell lines, with IC50 values of 23.04 nM and 15.65 nM, respectively (Fig. 2B and C). Furthermore, as shown in Figure S4, Pt@PCN-Cu demonstrated selective cytotoxicity against tumor cells within a specific concentration range, while sparing normal cells. This selectivity may be attributed to the consistently elevated metabolic activity and abnormally high copper levels in tumor cells, making them more susceptible to cuproptosis [26].

To further assess the antitumor effects of Pt@PCN-Cu, live-dead cell staining was performed on PANC-1 cells treated with various drugs. In the PBS-treated group, strong green fluorescence indicated live cells, whereas the Pt@PCN-Cu-treated group displayed robust red fluorescence, highlighting its potent cytotoxic effect (Fig. 2D). Additionally, APC-Annexin V/propidium iodide (PI) apoptosis assays were used to quantify apoptosis. The apoptotic rate in the Pt@PCN-Cu-treated group was 47.13%, 1.39-fold higher than that observed in the CuCl_2_ + ES-treated group (34.02%) (Fig. 2E). To further confirm the antitumor efficacy of Pt@PCN-Cu, a colony formation assay was conducted. The results demonstrated that the number of colonies in the Pt@PCN-Cu-treated group was reduced by approximately 70% compared to the PBS group (Fig. 2F). Overall, these findings underscore the potent cytotoxicity of Pt@PCN-Cu in vitro, highlighting its ability to efficiently kill tumor cells.

### Pt@PCN-Cu induces cuproptosis in vitro

**Fig. 3:**
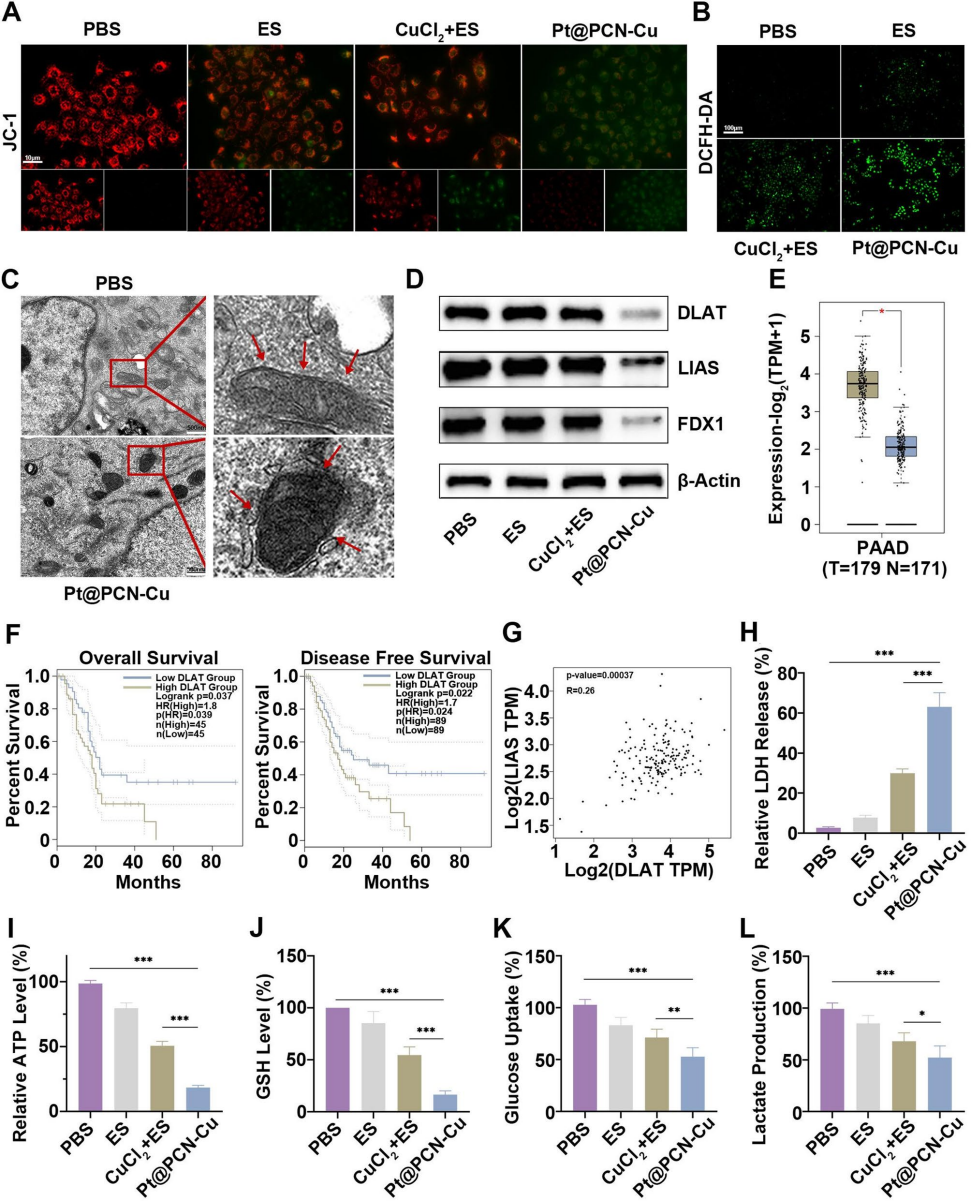
Pt@PCN-Cu induces robust intracellular ROS generation and severe mitochondrial damage, triggering cuproptosis. (**A**) CLSM images of PANC-1 cells stained with JC-1, illustrating changes in mitochondrial membrane potential following various treatments. Scale bar: 10 μm. (**B**) Representative CLSM images showing intracellular ROS generation in PANC-1 cells treated with different treatments. Scale bar: 100 μm. (**C**) TEM images of PANC-1 cells, highlighting mitochondrial morphology after PBS or Pt@PCN-Cu treatment. Scale bar: 500 nm. (**D**) Western blot analysis of DLAT, LIAS and FDX1 expression levels in PANC-1 cells after various treatment. (**E**) DLAT expression levels in PDAC tissues and normal pancreatic tissues analyzed using GEPIA2 (http://gepia2.cancer-pku.cn/). (**F**) Correlation between DLAT expression and OS and DFS in PDAC patients, as evaluated through GEPIA2. (**G**) Positive correlations between DLAT expression and the expression of LIAS in PDAC tumor tissues using GEPIA2. (**H**) LDH release assay quantifying membrane damage in PANC-1 cells subjected to the indicated treatments. (**I**) Measurement of intracellular ATP levels in PANC-1 cells after different treatments. (**J**) Measurement of intracellular GSH levels after various treatments. (**K**) Glucose consumption measured using a glucose assay kit. (**L**) Lactate production assessed using a lactate production detection kit. Data are presented as mean ± SD. Statistical analysis was performed using t-test or one-way ANOVA, with *P < 0.05, **P < 0.01 and ***P < 0.001

Previous findings indicated that copper ions released by Pt@PCN-Cu predominantly accumulate near the mitochondria (Fig. 1J). This suggests that Pt@PCN-Cu may exert its antitumor effects by inducing cuproptosis in tumor cells. Given the association between cuproptosis and mitochondrial damage, the mitochondrial membrane potential was evaluated using the JC-1 assay. As shown in Fig. 3A, the red-to-green fluorescence ratio was significantly decreased in PANC-1 cells treated with Pt@PCN-Cu, indicating a loss of mitochondrial membrane potential. Additionally, Pt@PCN-Cu treatment induced the strongest green fluorescence in PANC-1 cells, signifying elevated reactive oxygen species (ROS) levels (Fig. 3B). TEM was used to observe intracellular mitochondrial morphology. Pt@PCN-Cu-treated cells displayed substantial mitochondrial damage, characterized by mitochondrial shrinkage, increased membrane density, and a loss or complete absence of cristae (Fig. 3C). Notably, no signs of cell retraction, chromatin condensation, or apoptotic body formation were observed, providing further evidence that Pt@PCN-Cu induces cuproptosis rather than apoptosis. The expression levels of key genes associated with cuproptosis were also analyzed. DLAT, LIAS and FDX1 were significantly downregulated in Pt@PCN-Cu-treated cells (Fig. 3D). Notably, DLAT expression was found to be higher in PDAC tissues compared to normal pancreatic tissues (Fig. 3E). The correlation between DLAT expression and patient outcomes in PDAC was analyzed using GEPIA2 (Gene Expression Profiling Interactive Analysis 2) [27]. Patients with low DLAT expression exhibited significantly longer overall survival (OS) and disease-free survival (DFS) compared to those with high DLAT expression (Fig. 3F). Furthermore, the relationship between DLAT and other genes involved in lipoylation, including LIAS, FDX1, DLST and DLD, was explored. A positive correlation between DLAT expression and these genes was observed in PDAC tissues (Fig. 3G and Fig. S5). These findings suggest that Pt@PCN-Cu can effectively induce cuproptosis in tumor cells, highlighting its potential for clinical translation.

Recent studies have demonstrated that cuproptosis can promote immunogenic cell death, thereby enhancing the antitumor immune response [28]. In this study, Pt@PCN-Cu treatment significantly increased the release of intracellular lactate dehydrogenase (LDH), indicating that Pt@PCN-Cu-mediated cuproptosis effectively induces cell membrane damage (Fig. 3H). Similarly, the intracellular adenosine triphosphate (ATP) levels were significantly reduced in the Pt@PCN-Cu-treated group compared to the PBS group, indicating substantial ATP release into the extracellular environment following treatment (Fig. 3I). Since the cellular export of copper ions is highly dependent on elevated ATP levels, these findings also suggest that Pt@PCN-Cu facilitates the intracellular accumulation of copper ions. Meanwhile, intracellular GSH levels were assessed. Compared to the high GSH levels observed in the control group, the GSH content in PANC-1 cells was significantly reduced following treatment with Pt@PCN-Cu (Fig. 3J). Additionally, inhibition of tumor aerobic glycolysis has been shown to potentially promote cuproptosis [9]. To evaluate this effect, glucose consumption and lactate production were measured as indicators of glycolysis. The results revealed that Pt@PCN-Cu treatment significantly decreased glucose consumption and lactate production in PANC-1 cells (Fig. 3K and L). In summary, our findings demonstrated that Pt@PCN-Cu effectively induces cuproptosis in PANC-1 cells through the inhibition of glycolysis, ultimately leading to immunogenic cell death.

### Pt@PCN-Cu enhances PD-L1 expression through HK2-Dependent mechanism

**Fig. 4:**
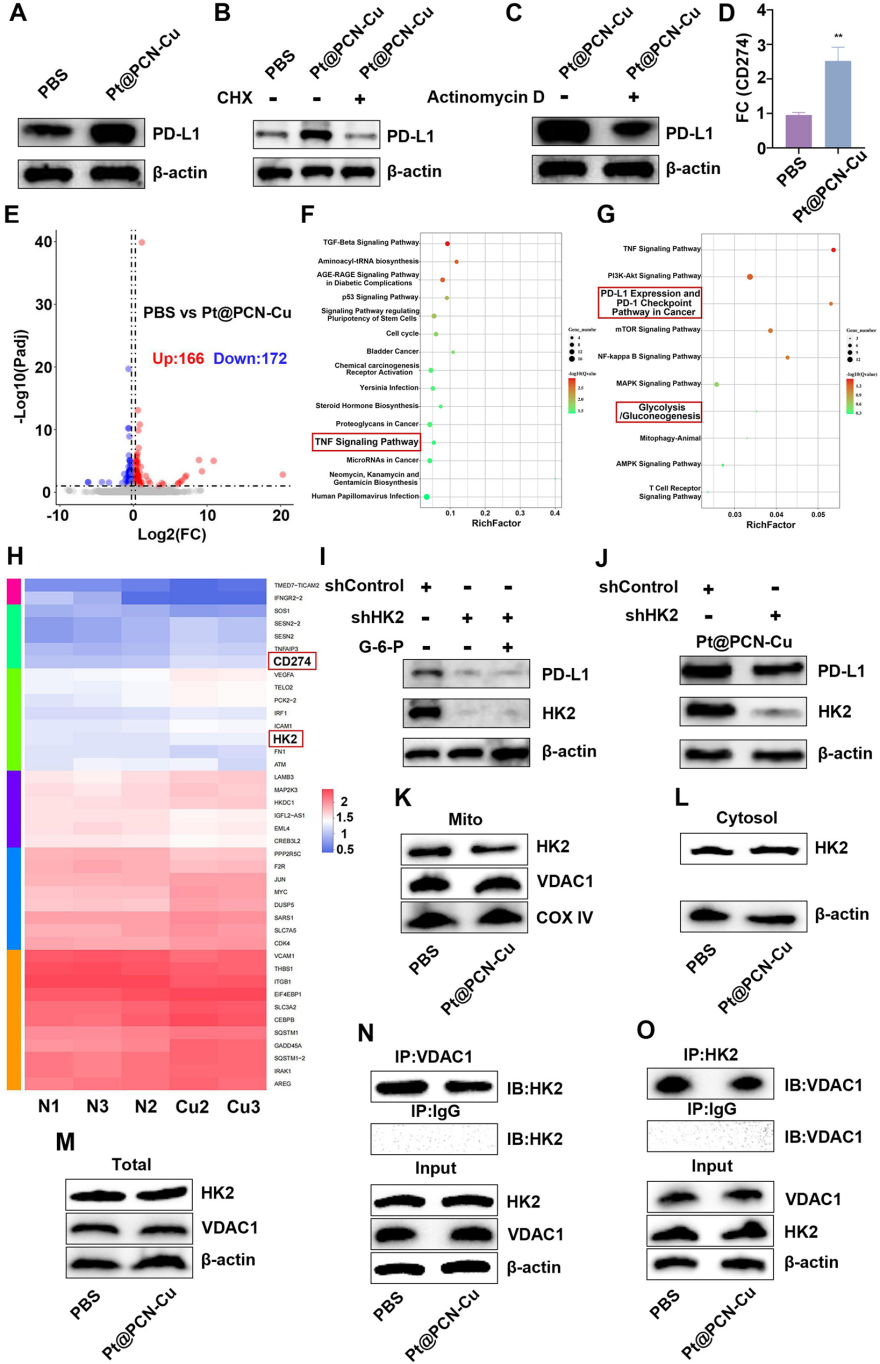
Pt@PCN-Cu enhances PD-L1 expression through an HK2-dependent mechanism. (**A**) PANC-1 cells were treated with Pt@PCN-Cu for 24 h and PD-L1 expression was assessed. (**B**) PANC-1 cells were treated with Pt@PCN-Cu for 24 h in the presence or absence of CHX. (**C**) PANC-1 cells were cultured with Pt@PCN-Cu for 24 h in the presence or absence of actinomycin D. (**D**) The qPCR analysis of CD274 mRNA expression. (**E**) Volcano plot showing differential gene expression between PBS group and Pt@PCN-Cu group. (**F**) Enrichment pathway analysis identifying the most significantly altered signaling pathways. (**G**) Enrichment pathway analysis highlighting key pathways. (**H**) Heatmap showing differentially expressed genes. (**I**) Western blot analysis of PD-L1 expression in PANC-1 cells stably expressing control shRNA or HK2 shRNA, treated with or without G-6-P for 12 h. (**J**) Western blot analysis of PD-L1 expression in PANC-1 cells stably expressing control shRNA or HK2 shRNA and cultured in the presence of Pt@PCN-Cu. (**K-M**) Mitochondrial and cytosolic fractions were isolated and western blot analysis was performed to evaluate HK2 and VDAC1 expression in each fraction. (**N**,** O**) Co-immunoprecipitation assay demonstrating the interaction between HK2 and VDAC1. Data are presented as mean ± SD. Statistical significance was determined using t-test, with **P < 0.01

Recent studies have suggested that intracellular copper levels in tumors can influence PD-L1 expression [29]. Moreover, an analysis of the correlation between DLAT and PD-L1 expression revealed a significant positive association in PDAC tissues (Fig. S6). To investigate the impact of Pt@PCN-Cu-mediated cuproptosis on PD-L1 expression, we assessed PD-L1 levels in PANC-1 cells. Our results demonstrated that Pt@PCN-Cu treatment significantly increased PD-L1 expression (Fig. 4A). This increase was attenuated by co-treatment with either cycloheximide (CHX) or actinomycin D, indicating that Pt@PCN-Cu regulates PD-L1 expression at both transcriptional and post-transcriptional levels (Fig. 4B and C). Consistent with these findings, Pt@PCN-Cu treatment also led to an increase in PD-L1 mRNA expression in PANC-1 cells (Fig. 4D). To elucidate the underlying mechanism by which Pt@PCN-Cu regulates PD-L1 expression, RNA sequencing (RNA-Seq) analysis was performed on PANC-1 cells treated with PBS or Pt@PCN-Cu. Differential expression analysis identified 338 genes with significant changes, including 166 upregulated and 172 downregulated genes, with HK2 among the upregulated targets (Fig. 4E, H and Fig. S7). Kyoto Encyclopedia of Genes and Genomes (KEGG) pathway analysis revealed that these differentially expressed genes were enriched in pathways related to the TNF signaling pathway and the PD-L1/PD-1 checkpoint pathway in cancer (Fig. 4F and G). Further analysis revealed a positive correlation between HK2 mRNA levels and PD-L1 mRNA levels in human PDAC specimens (Fig. S8A). GEPIA2 survival analysis demonstrated that elevated HK2 expression was negatively associated with OS and DFS in PDAC patients (Fig. S8B and C). Additionally, using the TIMER2.0 database, we observed that HK2 mRNA levels in PDAC specimens were inversely correlated with the infiltration of CD4⁺ and CD8⁺ T cells, as determined by TIMER and CIBERSORT algorithm analyses (Fig. S9).

Western blot analysis revealed that PD-L1 expression was reduced in PANC-1 cells following HK2 knockdown (Fig. 4I). Notably, this reduction could not be rescued by glucose-6-phosphate (G-6-P), suggesting that the glycolytic reactions downstream of HK2 are not involved in the regulation of PD-L1 expression (Fig. 4I). Consistently, HK2 depletion reduced PD-L1 expression in PANC-1 cells treated with Pt@PCN-Cu (Fig. 4J). Furthermore, we observed that Pt@PCN-Cu inhibited hexokinase activity in PANC-1 cells (Fig. S10). Given that Pt@PCN-Cu-induced cuproptosis leads to mitochondrial dysfunction and impaired glycolytic function, we hypothesize that Pt@PCN-Cu may alter the subcellular localization of HK2. To further investigate the impact of Pt@PCN-Cu on HK2 localization, we examined mitochondrial and cytoplasmic fractions. The expression of mitochondrial HK2 was significantly reduced, while cytoplasmic HK2 levels showed a slight increase (Fig. 4K-M). These results indicate that Pt@PCN-Cu induces the dissociation of HK2 from mitochondria, leading to a decrease in HK2 activity, while having minimal effects on cytoplasmic HK2. The voltage-dependent anion channel (VDAC), located in the mitochondrial outer membrane, serves as a gatekeeper for the transport of mitochondrial metabolites, mediating cross-talk between mitochondria and the rest of the cell [30]. VDAC1 binds to the N-terminal domain of HK2, anchoring HK2 to the mitochondrial outer membrane [31]. Co-immunoprecipitation analysis revealed a diminished binding capacity between HK2 and VDAC1 in the Pt@PCN-Cu-treated group, further supporting the role of Pt@PCN-Cu in disrupting the HK2-VDAC1 interaction (Fig. 4N and O). These findings collectively suggest that Pt@PCN-Cu upregulates PD-L1 expression through a mechanism dependent on HK2 modulation.

### Biosafety and biodistribution of Pt@PCN-Cu

**Fig. 5:**
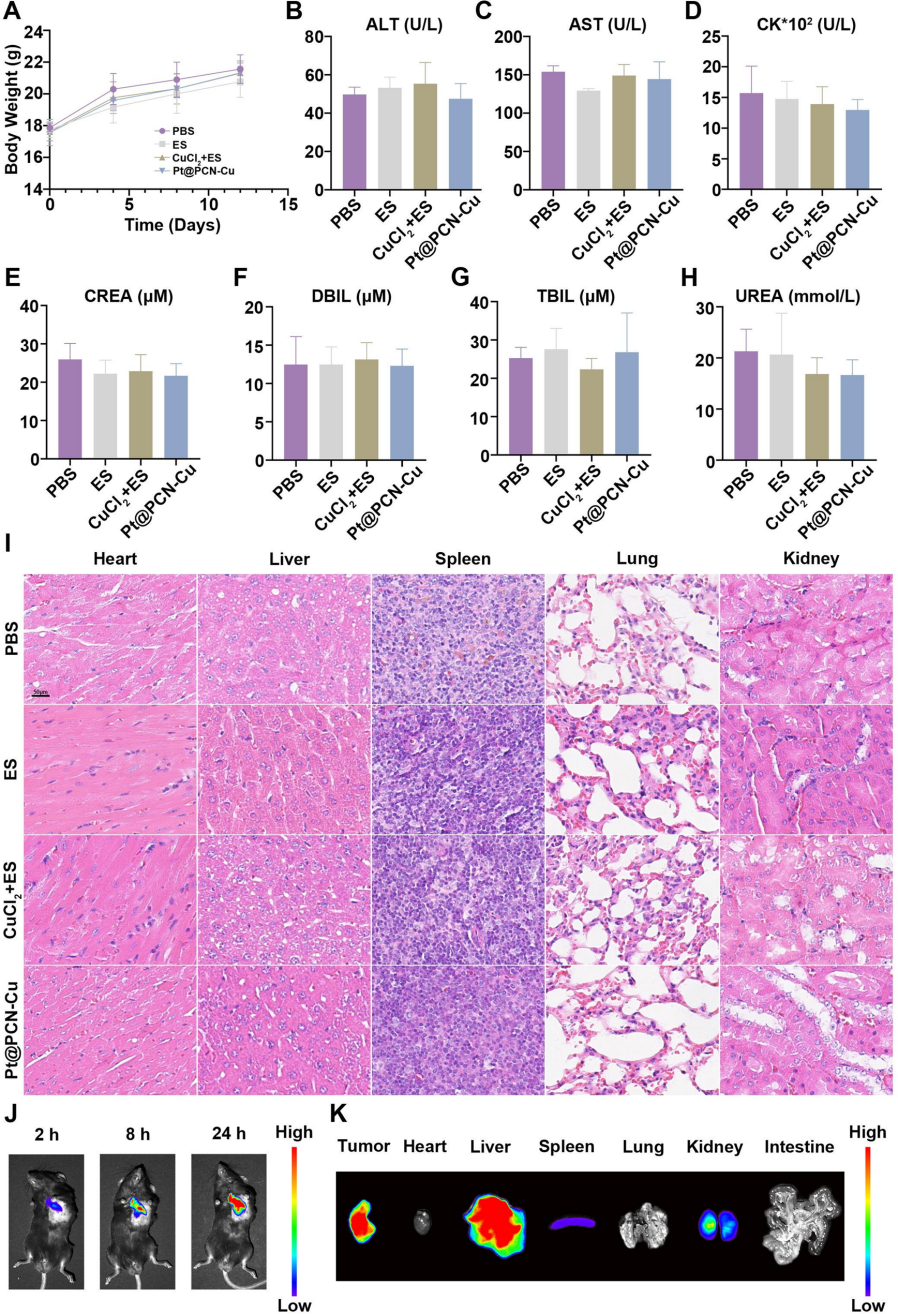
Biosafety and biodistribution of Pt@PCN-Cu. (**A**) Body weight changes in healthy C57BL/6 mice treated with PBS, ES, CuCl_2_ + ES and Pt@PCN-Cu, respectively. (**B-H**) Serum biochemical analysis of mice after various treatments: (**B**) ALT, (**C**) AST, (**D**) CK (**E**) Crea, (**F**) DBIL, (**G**) TBIL and (**H**) urea. (**I**) Histological assessment of major organs (heart, liver, spleen, lungs and kidneys) by H&E staining after various treatments. Scale bar: 50 μm. (**J**) In vivo biodistribution of Pt@PCN-Cu@Cy7.5. (**K**) Ex vivo imaging of tumors and major organs 24 h after administration of Pt@PCN-Cu@Cy7.5. Data are presented as mean ± SD

Given the significant antitumor activity of Pt@PCN-Cu observed in vitro, we further evaluated its therapeutic efficacy and biosafety in vivo. To assess the biosafety and biodistribution of Pt@PCN-Cu, healthy mice were administered PBS, ES (5 mg/kg), CuCl_2_ + ES (1 mg/kg + 5 mg/kg) and Pt@PCN-Cu (5 mg/kg) via tail vein injection for four consecutive treatments. On day 12, blood samples, major organs were collected from the treated mice for analysis. The results indicated that although there were slight variations in body weight among the different treatment groups, these changes were not statistically significant (Fig. 5A). Blood physiological and biochemical parameters, including alanine aminotransferase (ALT), aspartate aminotransferase (AST), creatine kinase (CK), serum creatinine (Crea), direct bilirubin (DBIL), total bilirubin (TBIL) and urea showed no significant differences between the Pt@PCN-Cu and PBS groups (Fig. 5B-H). Histopathological examination of major organs using hematoxylin and eosin (H&E) staining revealed no significant morphological changes in the Pt@PCN-Cu-treated group compared to the PBS group (Fig. 5I). In summary, Pt@PCN-Cu demonstrated low toxicity and excellent biosafety in vivo, supporting its potential for further therapeutic applications.

Next, an animal model of PDAC was established by subcutaneous injection of KPC cells to evaluate the biodistribution of Pt@PCN-Cu in vivo. Mice were injected with Pt@PCN-Cu@Cy7.5 via the tail vein, and fluorescence signals were monitored using an In Vivo Imaging System (IVIS). As shown in Fig. 5J, the Pt@PCN-Cu@Cy7.5 exhibited favorable tumor targeting capability due to the enhanced permeability and retention (EPR) effect. Fluorescence signals were detected at the tumor site as early as 2 h post-injection. Over time, the fluorescence intensity at the tumor site increased steadily, peaking at approximately 24 h, indicating rapid tumor accumulation and prolonged retention of Pt@PCN-Cu@Cy7.5 (Fig. 5J). After 24 h, the mice were sacrificed, and ex vivo analysis of biodistribution was performed. The ex vivo imaging demonstrated that Pt@PCN-Cu@Cy7.5 primarily accumulated at the tumor site, exhibiting limited distribution across other organs (Fig. 5K and Fig. S11). In addition, we also observed that Pt@PCN-Cu@Cy7.5 can accumulate extensively within tumor cells in vivo (Fig. S12). To further precisely evaluate the advantages of Pt@PCN-Cu, we measured the copper content in ex vivo tumor tissues using ICP-MS. The results confirmed that Pt@PCN-Cu achieved more effective Cu^2+^ delivery to tumor sites compared to other groups (Fig. S13). Furthermore, Cu^2+^ was highest in the tumors, significantly exceeding that in the liver, kidneys, heart, lungs, spleen, and intestines, indicating effective tumor targeting by Pt@PCN-Cu (Fig. S14). In summary, Pt@PCN-Cu demonstrated rapid targeting and accumulation at tumor sites, which enhances its potential for therapeutic applications.

### In vivo antitumor efficacy of Pt@PCN-Cu

**Fig. 6:**
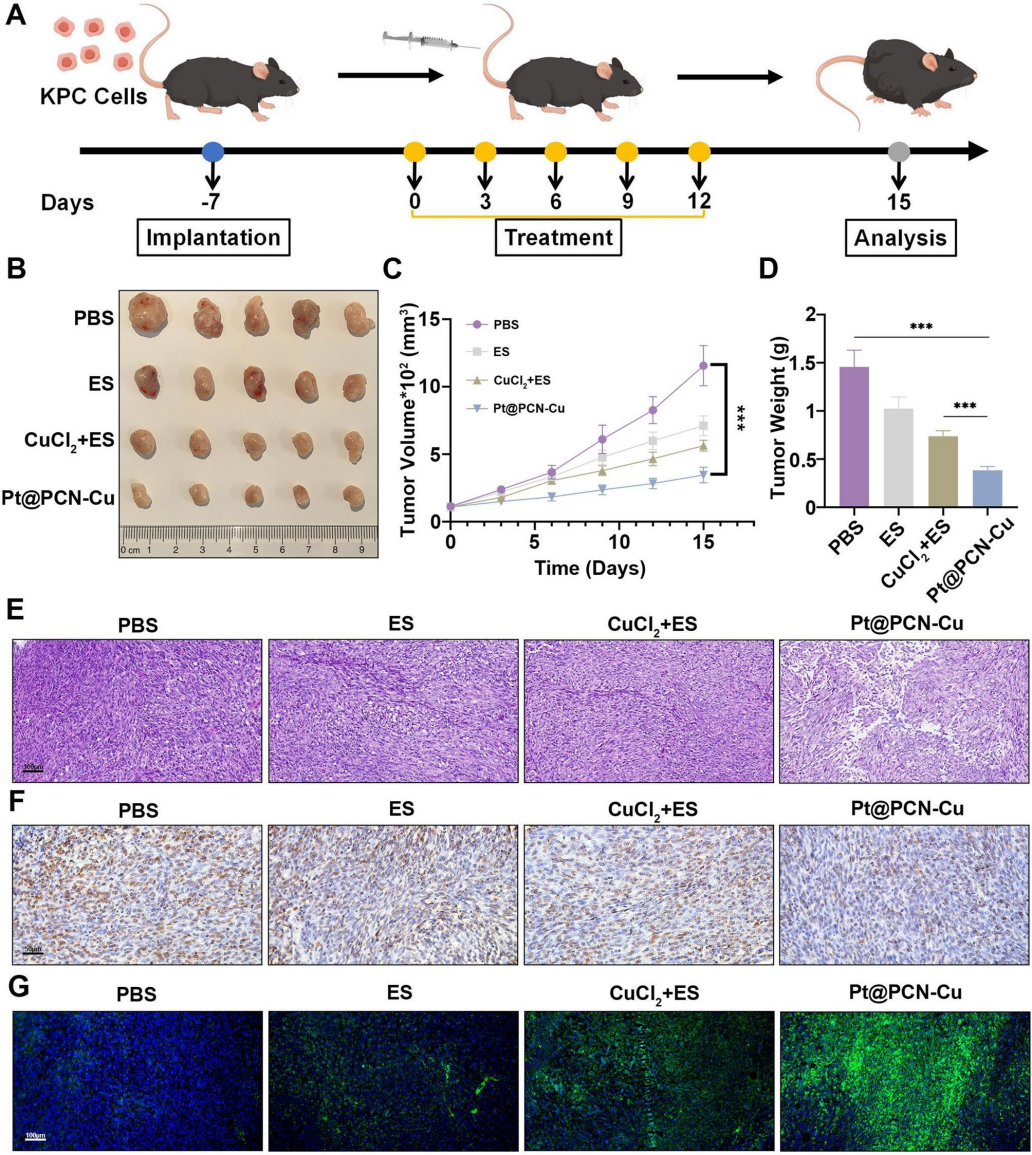
Pt@PCN-Cu activates antitumor responses in vivo. (**A**) Schematic representation of the treatment schedule, with arrows indicating the time points for intravenous drug administration. (**B**) Representative photographs of tumors excised from different treatment groups. (**C**) Tumor growth curves of mice subjected to different treatments. (**D**) Ex vivo tumor weights measured at the end of the study. (**E**,** F**) Representative images of H&E staining (**E**) and Ki67 staining (**F**) of tumor tissues from mice treated with different regimens. Scale bar: 100 μm–50 μm. (**G**) Immunofluorescence analysis of DLAT expression in tumors from different treatment groups. Scale bar: 100 μm. Data are presented as mean ± SD. Statistical significance was determined using a one-way ANOVA, with ***P < 0.001

A subcutaneous tumor model was established using KPC cells to evaluate the antitumor efficacy of Pt@PCN-Cu. Tumor-bearing mice were treated with PBS, ES, CuCl_2_ + ES or Pt@PCN-Cu via tail vein injection for five consecutive administrations. Tumor volumes were measured every three days from the first day of treatment (Fig. 6A). As shown in Fig. 6B, the ES and CuCl_2_ + ES groups exhibited limited tumor growth inhibition compared to the PBS group. In contrast, the Pt@PCN-Cu group demonstrated significantly enhanced tumor suppression compared to the other groups (Fig. 6C and D). This therapeutic effect was further confirmed by H&E staining, which revealed extensive nuclear fragmentation and nucleolysis in tumor tissues from the Pt@PCN-Cu group, indicating more effective tumor cell destruction (Fig. 6E). Immunohistochemical (IHC) staining for Ki67, a marker of cell proliferation, quantitatively confirmed the significant tumor-inhibitory effect of Pt@PCN-Cu (Fig. 6F and Fig. S15A). Furthermore, tumor tissues isolated from mice treated with Pt@PCN-Cu displayed the most intense DLAT fluorescence signal (green), suggesting a higher level of lipoylated protein aggregation (Fig. 6G and Fig. S15B). In summary, these results demonstrate that Pt@PCN-Cu exhibits superior tumor-suppressive activity, highlighting its potential as an effective therapeutic agent.

### Pt@PCN-Cu combined with αPD-L1 synergistically enhances antitumor efficacy

**Fig. 7:**
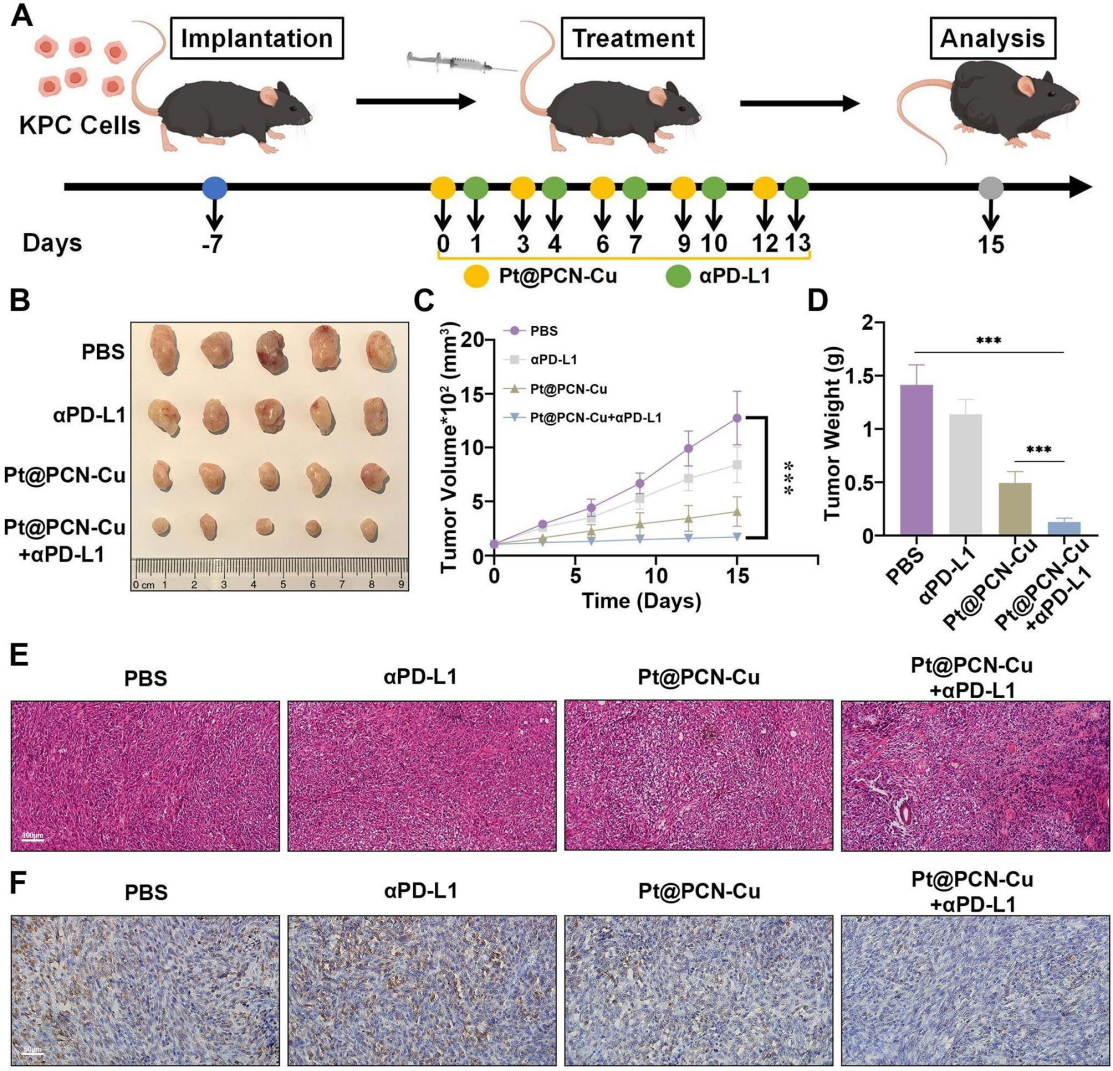
Combined treatment with Pt@PCN-Cu and αPD-L1 enhances in vivo antitumor efficacy. (**A**) Schematic representation of the treatment schedule, with arrows indicating the time points of intravenous injections. (**B**) Representative photographs of tumors excised from different treatment groups. (**C**) Tumor growth curves of mice subjected to different treatments. (**D**) Ex vivo tumor weights measured at the end of the study. (**E**,** F**) Representative images of H&E staining (**E**) and Ki67 staining (**F**) of tumor tissues from mice treated with different regimens. Scale bar: 100 μm–50 μm. Data are presented as mean ± SD. Statistical significance was determined using one-way ANOVA, with ***P < 0.001

Studies have demonstrated that the therapeutic efficacy of PD-L1 inhibitors is limited by the immunosuppressive TME, reducing the effectiveness of PD-L1 inhibitors when used alone [32]. In previous experiments, we found that Pt@PCN-Cu enhanced PD-L1 expression in PANC-1 cells, suggesting its potential to improve the application of PD-L1 inhibitors in PDAC (Fig. 4). Thus, combining Pt@PCN-Cu with PD-L1 inhibitor may represent a promising strategy for antitumor therapy. To investigate the therapeutic efficacy of Pt@PCN-Cu + αPD-L1, a subcutaneous tumor model using KPC cells was established (Fig. 7A). Tumor-bearing mice were treated intravenously with PBS, αPD-L1, Pt@PCN-Cu or Pt@PCN-Cu + αPD-L1 for five consecutive doses (Pt@PCN-Cu: 5 mg/kg; αPD-L1: 10 mg/kg). Tumor volumes were measured every three days starting from the first day of treatment. The results showed that tumor growth was significantly inhibited in the Pt@PCN-Cu + αPD-L1 group compared to the PBS, αPD-L1 and Pt@PCN-Cu groups (Fig. 7B). The tumor volumes were markedly smaller in the Pt@PCN-Cu + αPD-L1 group than in the other groups, and this trend was consistent with tumor weight measurements (Fig. 7C and D). Specifically, the observed inhibition rate of the combination therapy (90.95%) exceeded the sum of the individual effects of αPD-L1 (19.56%) and Pt@PCN-Cu (65.11%), which totaled 84.76%, suggesting a potential synergistic effect. Notably, this synergistic trend was significantly reduced in the shHK2 tumor model, as the observed inhibition rate of the combination therapy (72.55%) was only marginally higher than the additive effect of αPD-L1 (12.56%) and Pt@PCN-Cu (58.6%), which amounted to 71.16% (Fig. S16). The enhanced therapeutic effect of Pt@PCN-Cu + αPD-L1 was further confirmed by H&E staining, which revealed increased nuclear fragmentation and nucleolysis in the combination therapy group (Fig. 7E). Quantitative analysis of Ki67 staining confirmed the significant tumor-inhibitory efficacy of the combination treatment (Fig. 7F and Fig. S17). Importantly, the combined therapy significantly prolonged the survival of mice compared to the other treatment groups (Fig. S18). In summary, these findings demonstrate that Pt@PCN-Cu + αPD-L1 exhibits the strongest antitumor efficacy in vivo, highlighting its potential as an effective combination therapy for PDAC.

### Pt@PCN-Cu and αPD-L1 combined therapy effectively reprograms the TME

**Fig. 8:**
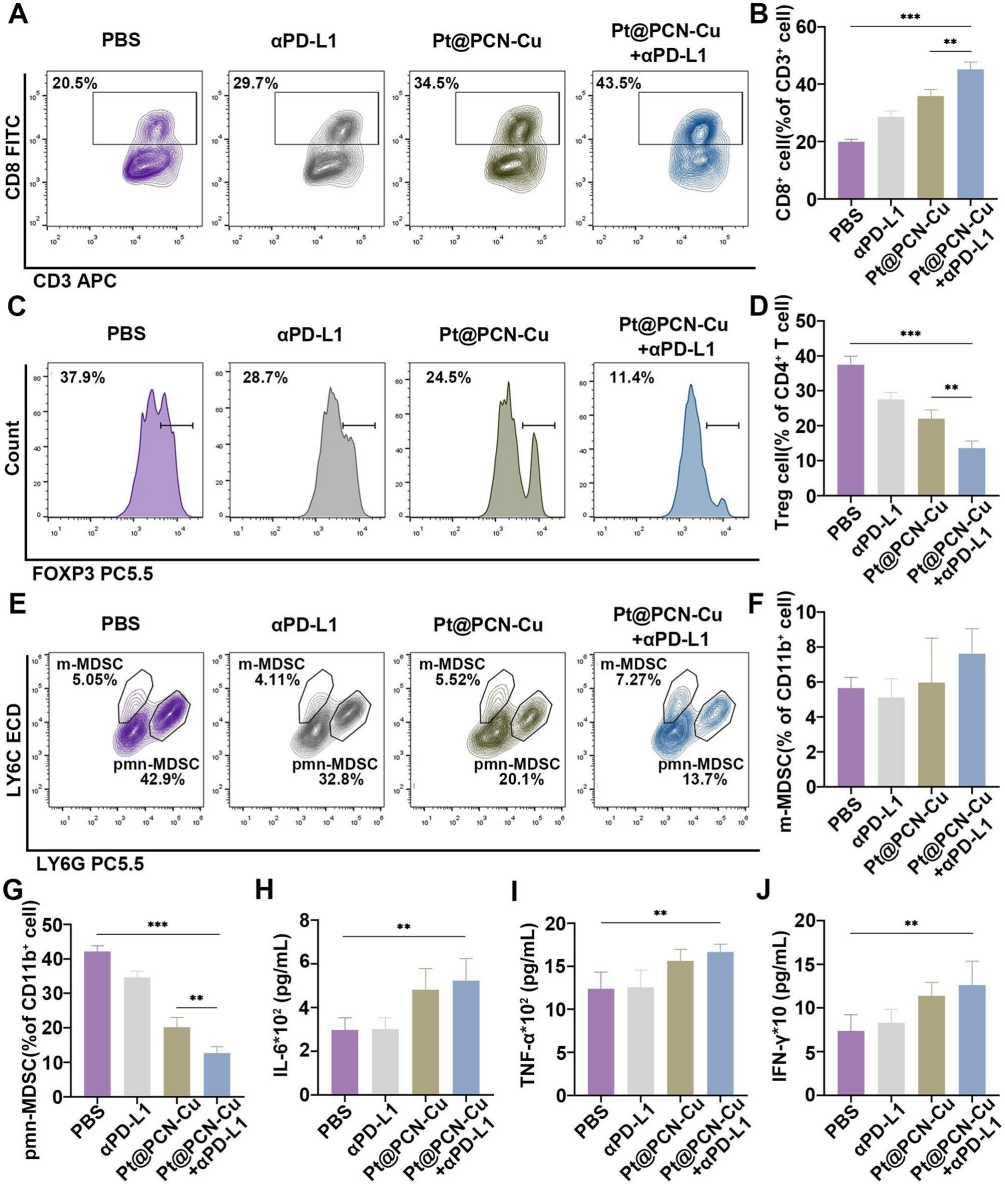
Evaluation of antitumor immunity induced by Pt@PCN-Cu and αPD-L1 combined therapy in vivo. (**A**,** B**) Representative flow cytometry plots and corresponding percentages of CD8⁺ T cells in tumor tissues from mice subjected to various treatments. (**C**,** D**) Representative flow cytometry plots and corresponding percentages of Tregs in tumor tissues from mice subjected to various treatments. (**E-G**) Representative flow cytometry plots and corresponding percentages of m-MDSCs and pmn-MDSCs in tumor tissues from mice subjected to various treatments. (**H-J**) Serum levels of inflammatory cytokines (IL-6, TNF-α and IFN-γ) measured using ELISAs. Data are presented as mean ± SD. Statistical significance was determined using a one-way ANOVA, with **P < 0.01 and ***P < 0.001

Accumulating evidence suggests that immune suppression within the TME poses a significant barrier to the clinical success of immunotherapies [33]. To evaluate the impact of Pt@PCN-Cu + αPD-L1 on the TME, tumor tissues were collected from mice treated with different regimens, and immune parameters related to the TME were analyzed (Fig. S19). Within the TME, excessive production of metabolic byproducts hinder T cell metabolic rewiring, thereby impairing antitumor immunity [34]. It is well established that CD8⁺ T cells play a pivotal role in mediating antitumor immunity [35]. As shown in Fig. 8A and B, CD8⁺ T cell infiltration was significantly higher in the Pt@PCN-Cu + αPD-L1 group compared to the PBS group. Tregs, characterized by the master transcription factor forkhead box protein P3 (Foxp3), play a crucial role in maintaining immune homeostasis and tolerance. Tregs can suppress the activation and differentiation of CD4⁺ helper T cells and CD8⁺ T cells, thereby impairing antitumor immunity [36]. Flow cytometry analysis of Treg proportions in tumor tissues revealed that the percentage of Tregs in the Pt@PCN-Cu + αPD-L1 group was 11.4%, only one-third of that in the PBS group (Fig. 8C and D). These findings indicate that Pt@PCN-Cu + αPD-L1 effectively reduces Treg levels within tumor tissues, alleviating the immunosuppressive TME.

TAMs are macrophages that infiltrate tumors, with activated macrophages primarily classified into M1 and M2 macrophages [37]. M1 macrophages enhance T cell effector functions by secreting pro-inflammatory cytokines and chemokines, as well as presenting antigens. In contrast, M2 macrophages exhibit immunosuppressive properties, inhibiting the proliferation of effector T cells. The polarization of M1 macrophages into M2 macrophages within the TME is a key contributor to tumor immunosuppression and immune evasion, ultimately leading to poor cancer therapy outcomes and unfavorable prognoses [38]. To investigate whether Pt@PCN-Cu + αPD-L1 could induce the repolarization of M2 macrophages to M1 macrophages within the TME, we analyzed the M1/M2 macrophage ratio in TAMs from mice treated with different drugs. Notably, tumors from mice treated with Pt@PCN-Cu + αPD-L1 exhibited a significantly higher number of M1 macrophages compared to those treated with other drugs (Figure S20A). Conversely, the number of M2 macrophages was significantly lower in Pt@PCN-Cu + αPD-L1-treated tumors than in those treated with other drugs (Figure S20B). Furthermore, the M1/M2 ratio in tumors from Pt@PCN-Cu + αPD-L1-treated mice reached 1.85, which was 3.8 times higher than that observed in PBS-treated mice (Figure S21). These findings collectively suggest that Pt@PCN-Cu + αPD-L1 effectively induces the repolarization of TAMs from the M2 phenotype to the M1 phenotype.

MDSCs are critical components of the immunosuppressive TME and are frequently associated with tumor cell survival and drug resistance. Based on their morphology and phenotype, MDSCs are commonly classified into monocytic MDSCs (m-MDSCs) and polymorphonuclear MDSCs (pmn-MDSCs), both characterized by their immunosuppressive functions [39, 40]. To evaluate the impact of Pt@PCN-Cu + αPD-L1 on MDSCs within the TME, the relative proportions of MDSC subtypes in tumor tissues were analyzed. The proportion of pmn-MDSCs in tumors from mice treated with Pt@PCN-Cu + αPD-L1 was significantly reduced to 13.7%, approximately one-third of the level observed in tumors from PBS-treated mice (Fig. 8E and G). However, no significant differences were observed in the proportion of m-MDSCs between treatment groups (Fig. 8E and F). Furthermore, levels of key proinflammatory cytokines, including interleukin-6 (IL-6), tumor necrosis factor-α (TNF-α) and interferon-γ (IFN-γ), were markedly elevated in mice treated with Pt@PCN-Cu + αPD-L1, indicating enhanced immune activation (Fig. 8H-J). Together, these results demonstrate that Pt@PCN-Cu combined with αPD-L1 immunotherapy effectively reprograms the TME, enhancing antitumor immunity and reducing immunosuppression.

## Discussion

Cell death plays a pivotal role in regulating tumor therapeutic responses. The modes of cell death comprise apoptosis, autophagy, ferroptosis and necrosis, have been extensively studied in cancer biology [41]. Recently, a novel form of regulated cell death, termed cuproptosis, has garnered considerable attention. Unlike other forms of regulated cell death, cuproptosis is characterized by the accumulation of lipid peroxides and is not inhibited by the inhibitors targeting the aforementioned cell death pathways [9]. Exploiting the cytotoxic effects of copper, particularly in cancer cells with dysregulated copper homeostasis, offers a promising strategy for developing targeted therapies and has the potential to enhance the efficacy of existing treatment modalities. Although cuproptosis holds significant promise for cancer therapy, the glycolysis-dominated metabolic pattern and the tight regulation of intracellular copper levels by ATP7B in cancer cells limit the occurrence of cuproptosis [10]. Copper ionophores, small molecules, and nanomedicine have emerged as essential tools for inducing cuproptosis in cancer cells, offering potential solutions to overcome the above obstacles [42]. While copper ionophores and small molecules can effectively modulate intracellular copper levels to trigger mitochondrial dysfunction and oxidative stress, their clinical application is often limited by poor stability and off-target effects [43]. In contrast, nanomedicine offers significant advantages, including precise delivery of copper ions or complexes to tumor tissues and controlled release [44]. However, studies in this area remain scarce, highlighting the need for further exploration. In our research, Pt@PCN-Cu exhibits excellent physicochemical properties and remarkable cascade catalytic activity, providing a solid foundation for further in vitro and in vivo. Structurally, Pt@PCN-Cu features a uniform spindle-shaped morphology, outstanding dispersibility, and exceptional stability, as demonstrated in Fig. 1. Within the system, Cu^2+^ react with GSH, generating highly toxic Cu⁺ and GSSG (Fig. S3). Once internalized, Pt@PCN-Cu triggers copper release through cascade catalytic reactions (Fig. 1H and I). The released copper selectively accumulates in mitochondria, resulting in a significant inhibition of PANC-1 cell proliferation (Figs. 1J and 2). These key properties of NPs are primarily attributed to their cationic surface charge, facilitating strong interactions with anionic cellular membranes [7]. However, unlike conventional cationic NPs that often exhibit increased toxicity, Pt@PCN-Cu has demonstrated relatively greater safety (Fig. S2 and S4). Furthermore, Pt@PCN-Cu induces cuproptosis primarily through mitochondrial dysfunction, as evidenced by glycolysis inhibition, which ultimately triggers immunogenic cell death (Fig. 3). Moreover, Pt@PCN-Cu exhibited excellent biosafety in vivo and achieved superior tumor inhibition compared to CuCl_2_ + ES (Figs. 5 and 6). The nature-inspired properties of Pt@PCN-Cu offer distinct advantages over conventional nanomaterials, underscoring its potential as a promising candidate for further exploration in biomedical applications.

Although αPD-L1 therapy has demonstrated considerable clinical efficacy in various cancers, its effectiveness in PDAC remains limited [45, 46], primarily due to the heterogeneity of the TME and the complexity of immune evasion mechanisms. A major contributing factor is that cancer cells can downregulate PD-L1 expression through various mechanisms, thereby hindering the effective binding and action of PD-L1 inhibitors [47]. Consequently, personalized therapeutic strategies aimed at regulating PD-L1 expression could offer a promising approach to enhancing the efficacy of PD-L1 inhibitors in PDAC. Recent studies have implicated copper ions in the regulation of PD-L1 expression [48–51]. However, the relationship between copper and PD-L1 expression remains controversial, prompting questions regarding the consistency of this association. Moreover, the specific molecular pathways linking copper to the regulation of PD-L1 remain unclear, highlighting the need for further research to elucidate these mechanisms and their potential impact on PDAC immunotherapy. In our study, Pt@PCN-Cu was found to upregulate PD-L1 expression through an HK2-dependent mechanism (Fig. 4A-J). HK2 is known to bind to VDAC on the mitochondrial outer membrane, enhancing its functional activity [18]. Our findings suggest that Pt@PCN-Cu promotes the dissociation of HK2 from VDAC1, facilitating its translocation to the cytoplasm and subsequently upregulating PD-L1 expression (Fig. 4K-O). This explains how Pt@PCN-Cu + αPD-L1 enhance the antitumor effects. In spite of this, the precise mechanisms underlying the translocation of HK2 to the nucleus and its regulation of PD-L1 expression remain unclear, warranting further investigation. Although several studies have reported a correlation between elevated HK2 expression and increased PD-L1 levels [15, 52], the dissociation of HK2 from mitochondria, induced by cuproptosis, may represent a critical mechanism for overcoming αPD-L1 resistance through modulation of PD-L1 expression.

Enhancing the sensitivity of PDAC to αPD-L1 therapy is essential for improving the efficacy of immunotherapy in this challenging cancer type [8]. However, studies investigating the combination of cuproptosis and αPD-L1 therapy in PDAC are scarce. Copper-based nanomaterials, exploiting the EPR effect, have demonstrated potential to synergize with immunotherapy, thereby enhancing antitumor efficacy [53]. In this study, our combination strategy exhibited superior therapeutic effects compared to monotherapy (Fig. 7). Moreover, Pt@PCN-Cu-mediated therapy reprogrammed the TME, thereby enhancing the tumor’s responsiveness to immunotherapy (Fig. 8). Consequently, the combination of Pt@PCN-Cu and αPD-L1 therapy led to significant therapeutic improvements in PDAC. Traditional combination therapies involving αPD-L1 in clinical settings are often associated with significant side effects [54]. In contrast, Pt@PCN-Cu + αPD-L1 therapy emerges as a promising alternative, demonstrating enhanced therapeutic efficacy while mitigating the risk of adverse effects. This innovative approach holds significant potential for improving PDAC treatment outcomes.

The findings of this study should be interpreted considering certain limitations. First, although in vitro studies and subcutaneous PDAC mouse models were utilized, these models do not fully replicate the complex tumor microenvironment and immune suppression characteristic of human PDAC. The incorporation of an orthotopic mouse tumor model could strengthen the reliability and translational relevance of this study. Second, the precise mechanisms underlying the translocation of HK2 to the nucleus and its regulation of PD-L1 expression remain unclear, necessitating further investigation to establish the direct mechanistic link between HK2 dissociation from mitochondria and PD-L1 upregulation. Finally, although the combination of Pt@PCN-Cu and αPD-L1 demonstrated potent antitumor efficacy in vivo, the precise nature of their interaction remains to be fully elucidated. To address this critical gap, further mechanistic investigations, including conditional knockout models and other targeted approaches, are warranted.

## Conclusions

**Fig. 9:**
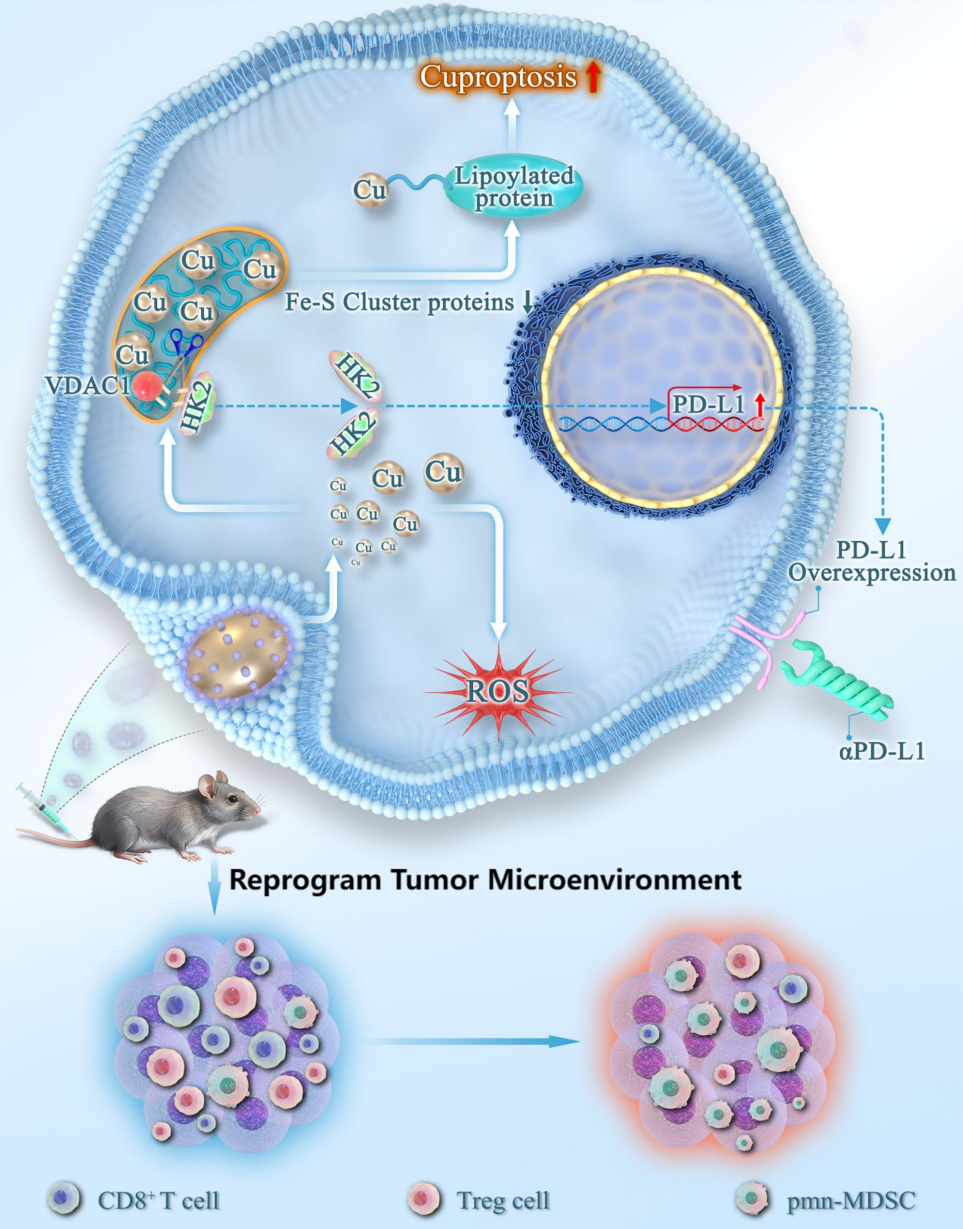
Enhanced cancer therapy by targeting cancer cells through Pt@PCN-Cu-induced cuproptosis combined with αPD-L1.

In summary, we developed a nanozyme, Pt@PCN-Cu, designed to sensitize cancer cells to cuproptosis and activate cuproptosis-mediated immunotherapy for PDAC. Specifically, Pt@PCN-Cu effectively promotes intracellular copper accumulation, inducing cuproptosis, and upregulates PD-L1 expression through an HK2-dependent mechanism in PDAC cells. Furthermore, Pt@PCN-Cu-induced cuproptosis, in combination with αPD-L1 therapy, synergistically enhances antitumor immunity, offering a promising and potent strategy for PDAC treatment (Fig. 9).

## Electronic supplementary material

Below is the link to the electronic supplementary material.

Supplementary Material 1

Supplementary Material 2

## Data Availability

No datasets were generated or analysed during the current study.

## Abbreviations

PDAC: Pancreatic ductal adenocarcinoma
ICIs: Immune checkpoint inhibitors
αPD-L1: Anti-programmed cell death ligand-1
TAMs: Tumor-associated macrophages
Tregs: Regulatory T cells
MDSCs: Myeloid-derived suppressor cells
CD8⁺ T cell: CD8⁺ cytotoxic T lymphocyte
TME: Tumor microenvironment
LIAS: Lipoyl synthase
FDX1: Ferredoxin 1
DLAT: Dihydrolipoamide S-acetyltransferase
HK2: Hexokinase 2
GSH: Glutathione
GSSG: Oxidized glutathione
ER: Endoplasmic reticulum
ES: Elesclomol
ROS: Reactive oxygen species
OS: Overall survival
DFS: Disease-free survival
LDH: Lactate dehydrogenase
ATP: Adenosine triphosphate
CHX: Cycloheximide
G-6-P: Glucose-6-phosphate
VDAC: Voltage-dependent anion channel
ALT: Alanine aminotransferase
AST: Aspartate aminotransferase
CK: Creatine kinase
TBIL: Total bilirubin
DBIL: Direct bilirubin
Foxp3: Forkhead box protein P3
pmn-MDSCs: polymorphonuclear MDSCs
m-MDSCs: monocytic MDSCs
IL-6: Interleukin-6
TNF-α: Tumor necrosis factor-α
IFN-γ: Interferon-γ
