# Supplementary figures and images for: Novel Pt@PCN-Cu-induced cuproptosis amplifies αPD-L1 immunotherapy in pancreatic ductal adenocarcinoma through mitochondrial HK2-mediated PD-L1 upregulation
Source: J Exp Clin Cancer Res. 2025 May 17;44:149. doi: 10.1186/s13046-025-03409-4 (PMC12085017; doi:10.1186/s13046-025-03409-4)

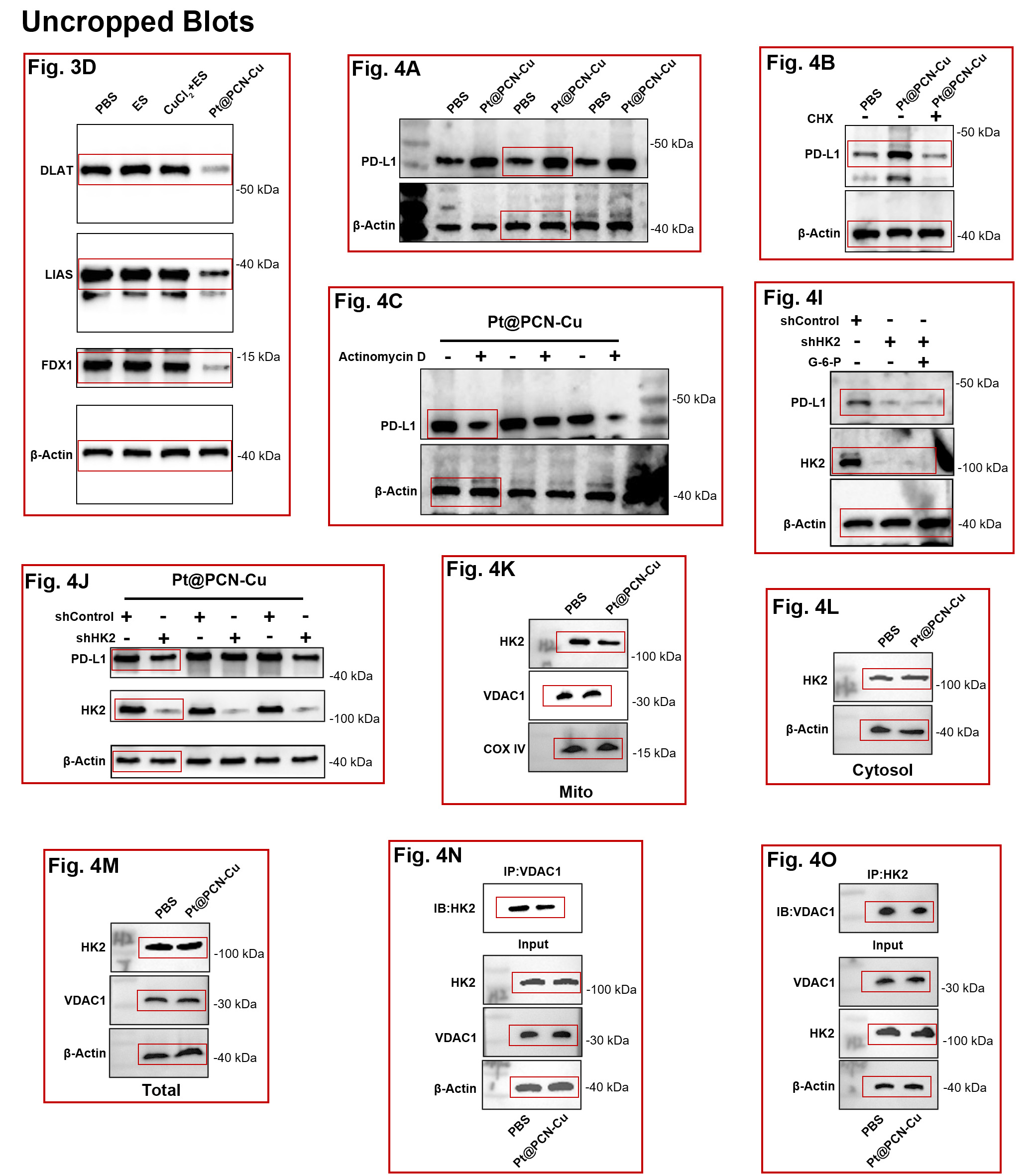

Supplement: Supplementary file 1 — Supplementary Material 1 [file 13046_2025_3409_MOESM1_ESM.jpg]
