# Supplementary material for: Novel Pt@PCN-Cu-induced cuproptosis amplifies αPD-L1 immunotherapy in pancreatic ductal adenocarcinoma through mitochondrial HK2-mediated PD-L1 upregulation
Source: J Exp Clin Cancer Res. 2025 May 17;44:149. doi: 10.1186/s13046-025-03409-4 (PMC12085017; doi:10.1186/s13046-025-03409-4)
Supplement: Supplementary file 2 — Supplementary Material 2 [file 13046_2025_3409_MOESM2_ESM.doc]

**Supplementary Information**

**Novel Pt@PCN-Cu-Induced Cuproptosis Amplifies αPD-L1 Immunotherapy in Pancreatic Ductal Adenocarcinoma through Mitochondrial HK2-Mediated PD-L1 Upregulation**

Pengyu Wang^1†^, Weihua Guo^1†^, Shuyue Liu^1†^, Shouyi Li^1†^, Jiaqi Li^1^, Bowen Ding^1^, Fengyi Yin^1^, Yang Yang^1^, Xingjiang Li^2^, Pei Cao^1^, Chaozhe Ma^1^, Wanying Zhang^2^, Yidan Song^2^, Yating Geng^2^, Lantao Liu^2*^, Jing Hu^3*^, Jihui Hao^1*^, Yukuan Feng^1*^

^1^ Pancreas Center, Tianjin Medical University Cancer Institute and Hospital, National Clinical Research Center for Cancer, State Key Laboratory of Druggability Evaluation and Systematic Translational Medicine, Tianjin Key Laboratory of Digestive Cancer, Tianjin’s Clinical Research Center for Cancer, Tianjin 300060, China.

^2^ School of Basic Medicine, Mudanjiang Medical University, Mudanjiang 157011, China.

^3^ School of Basic Medicine, Tianjin Medical University, Tianjin 300070, China.

†These authors made equal contributions to this work.

*Corresponding authors.

E-mail addresses: fengyukuan@tjmuch.com (Y.F.); [haojihui@tjmuch.com](mailto:haojihui@tjmuch.com) (J.H); jinghu_2001@hotmail.com (J.H); liulantao@mdjmu.com (L.L)


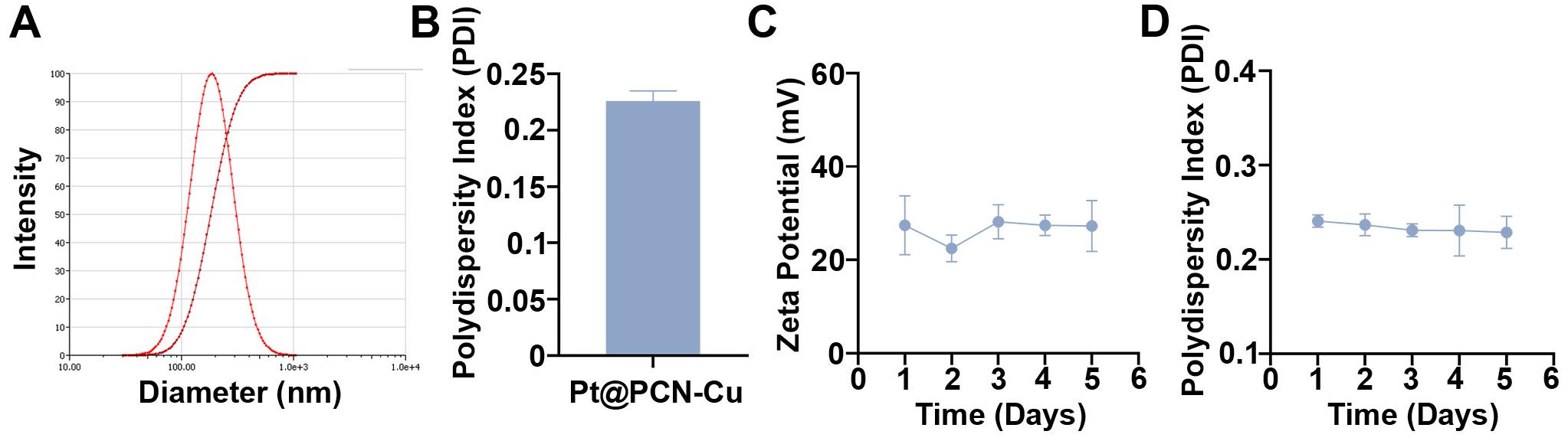


****Fig. S1**** (**A**) Particle size distribution of Pt@PCN-Cu in water as measured by dynamic light scattering (DLS). (**B**) Polydispersity index (PDI) of Pt@PCN-Cu. (**C, D**) Changes in zeta potential and PDI of the supernatant from Pt@PCN-Cu solutions in PBS, observed after five days of storage.


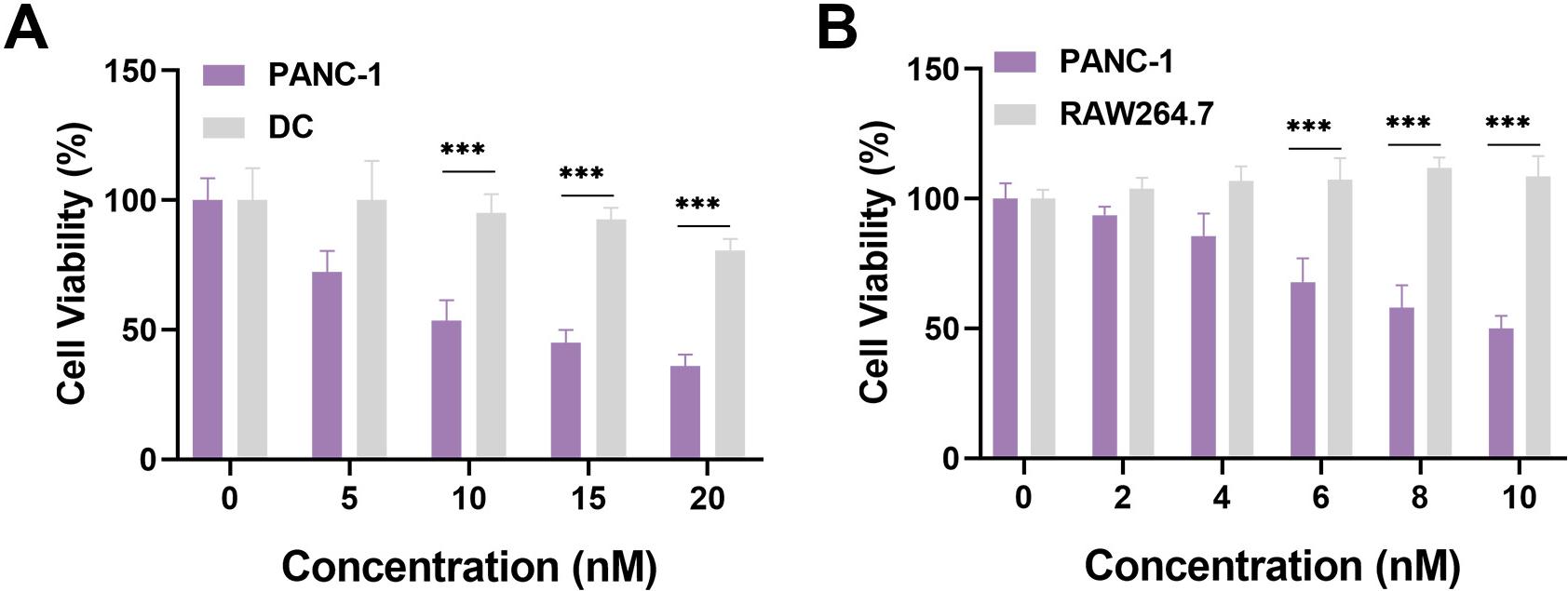


**Fig S2** (**A**) Viabilities of PANC-1 cells and DCs after treatment with different concentrations of Pt@PCN-Cu. (**B**) Viabilities of PANC-1 and RAW264.7 cells after treatment with different concentrations of Pt@PCN-Cu. Data are presented as mean±SD. Statistical significance was determined using t-test, with ****P*<0.001.


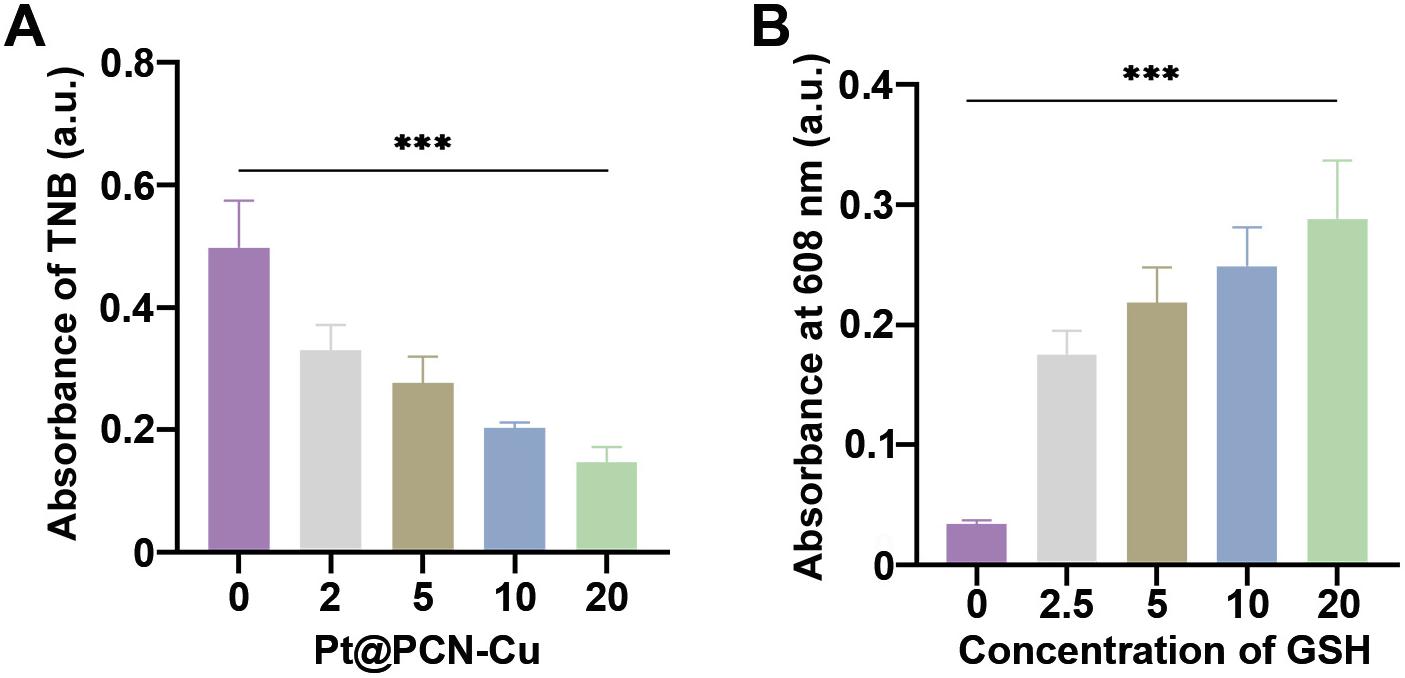


**Fig. S3** (**A**) Absorbance of TNB solutions at 412 nm after treatment with different concentrations of Pt@PCN-Cu. (**B**) Evaluation of GSH-triggered generation of Cu^+^. Data are presented as mean±SD. Statistical analysis was performed using one-way ANOVA, with ****P*<0.001.


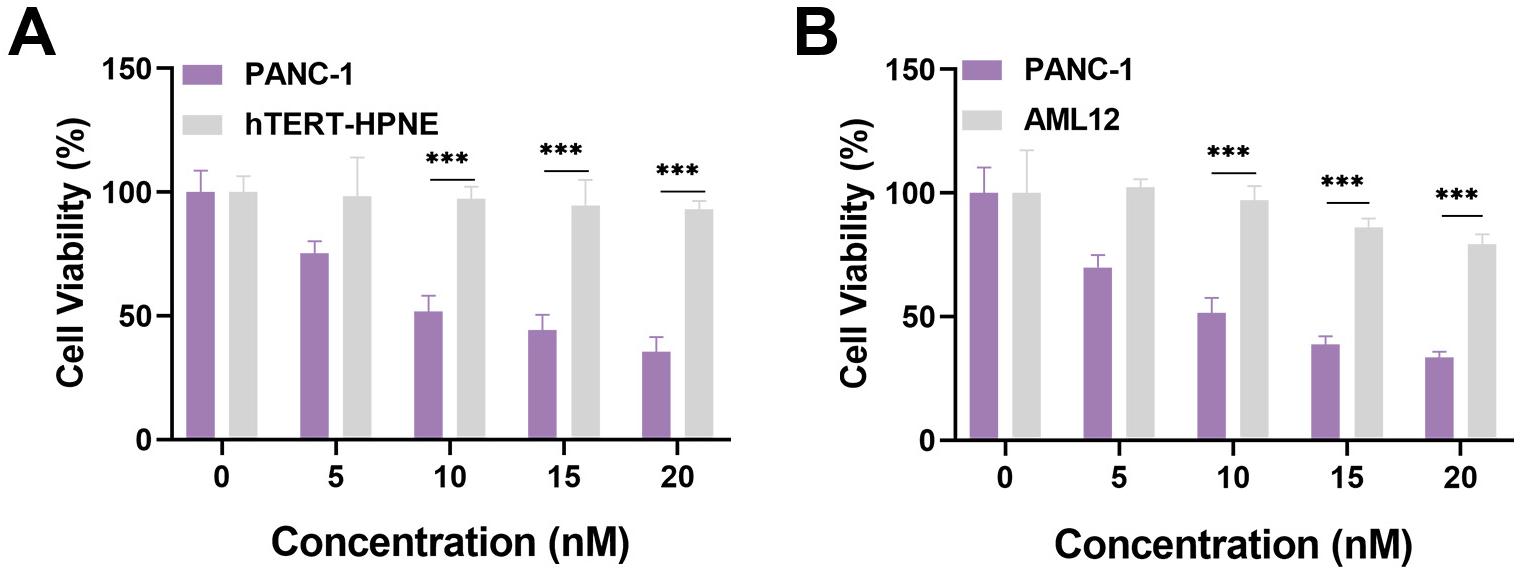


**Fig S4** (**A**) Viabilities of PANC-1 and hTERT-HPNE cells after treatment with different concentrations of Pt@PCN-Cu. (**B**) Viabilities of PANC-1 and AML12 cells after treatment with different concentrations of Pt@PCN-Cu. Data are presented as mean±SD. Statistical significance was determined using t-test, with ****P*<0.001.


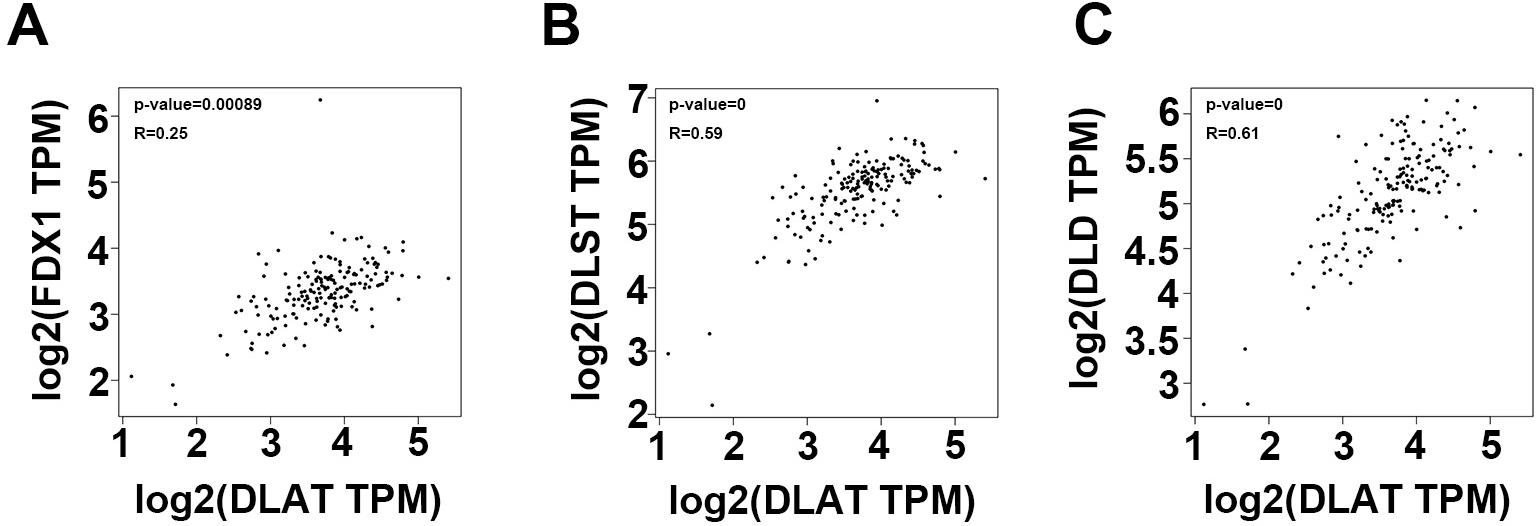


****Fig. S5**** (**A-C**) Positive correlations between DLAT expression and the expression of (**A**) FDX1, (**B**) DLST and (**C**) DLD in PDAC tumor tissues.


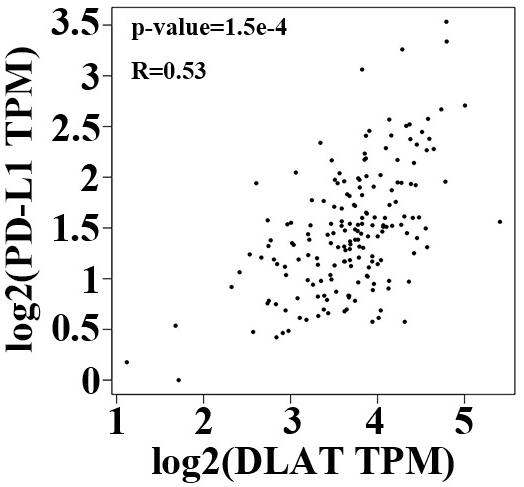


****Fig. S6**** Positive correlation observed between DLAT expression and PD-L1 expression in tumor tissues of PDAC.


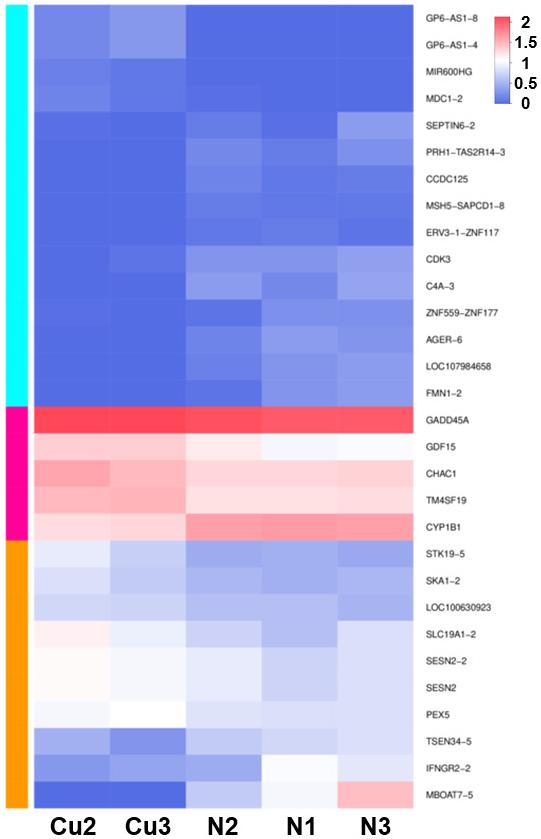


****Fig. S7**** Heatmap showing differentially expressed genes.


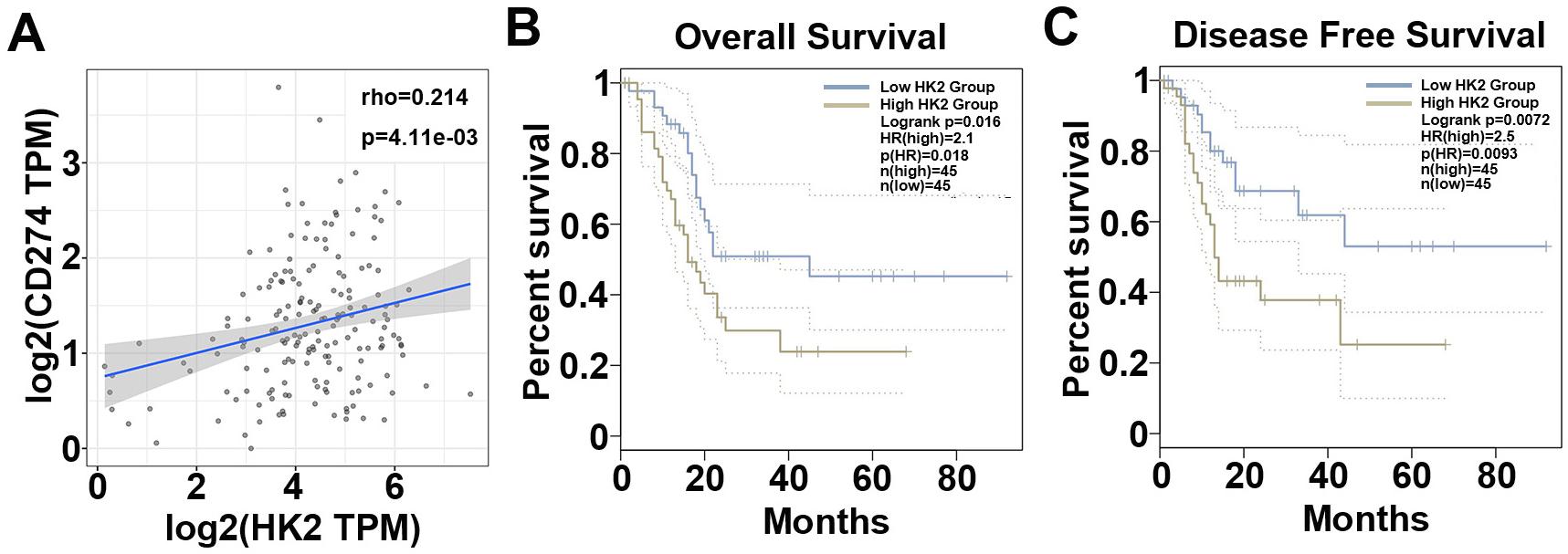


****Fig. S8**** (**A**) Correlation analysis between CD274 mRNA and HK2 mRNA expression in PDAC samples, analyzed using TIMER2.0 database, with the Spearman’s rho value presented. (**B, C**) Association between HK2 expression levels and (**B**) OS and (**C**) DFS in PDAC patients, using GEPIA2.


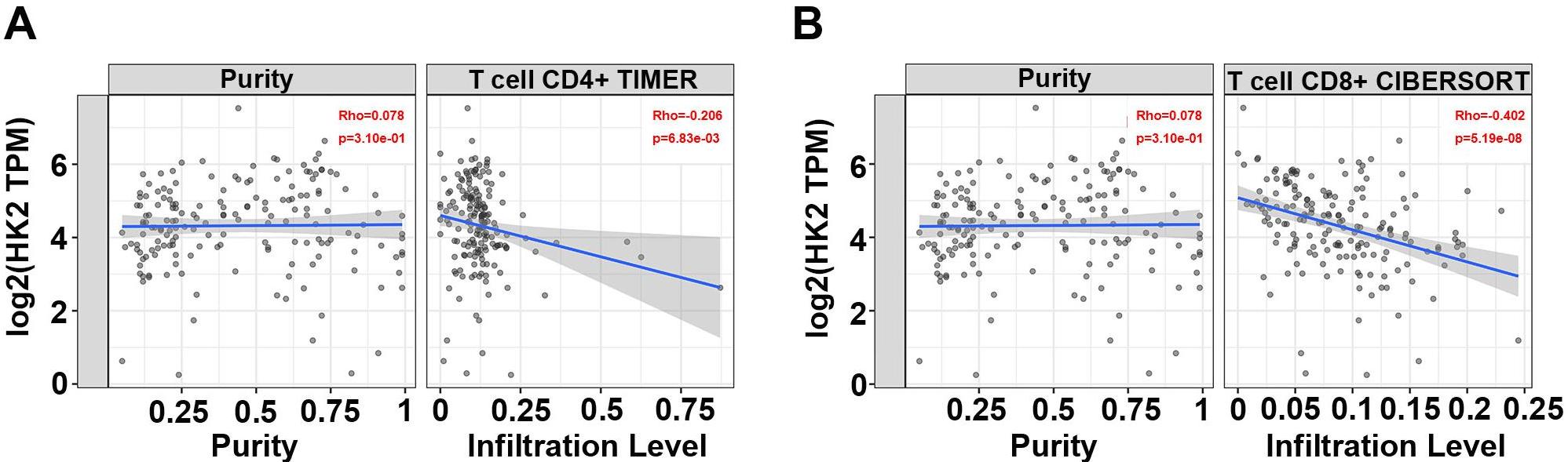


****Fig. S9**** (**A**) Correlation between HK2 mRNA expression levels and CD4⁺ T cell infiltration in PDAC patient specimens, analyzed using the TIMER algorithm in the TIMER2.0 database, with the Spearman’s rho value presented. (**B**) Correlation between HK2 mRNA expression levels and CD8⁺ T cell infiltration in PDAC patient specimens, analyzed using the CIBERSORT algorithm in the TIMER2.0 database, with the Spearman’s rho value presented.


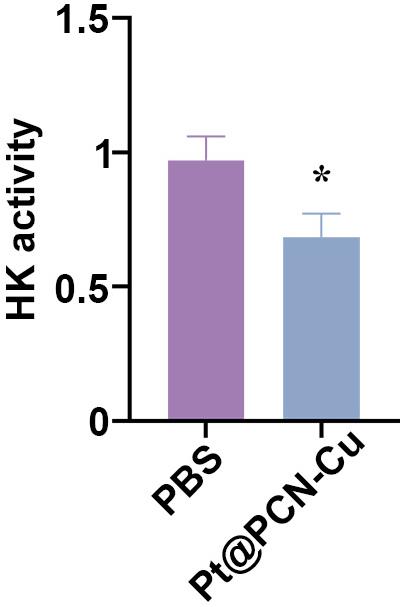


****Fig. S10**** HK activity measured using the hexokinase activity detection kit. Data are presented as mean±SD. Statistical significance was determined using t-test, with **P*<0.05.


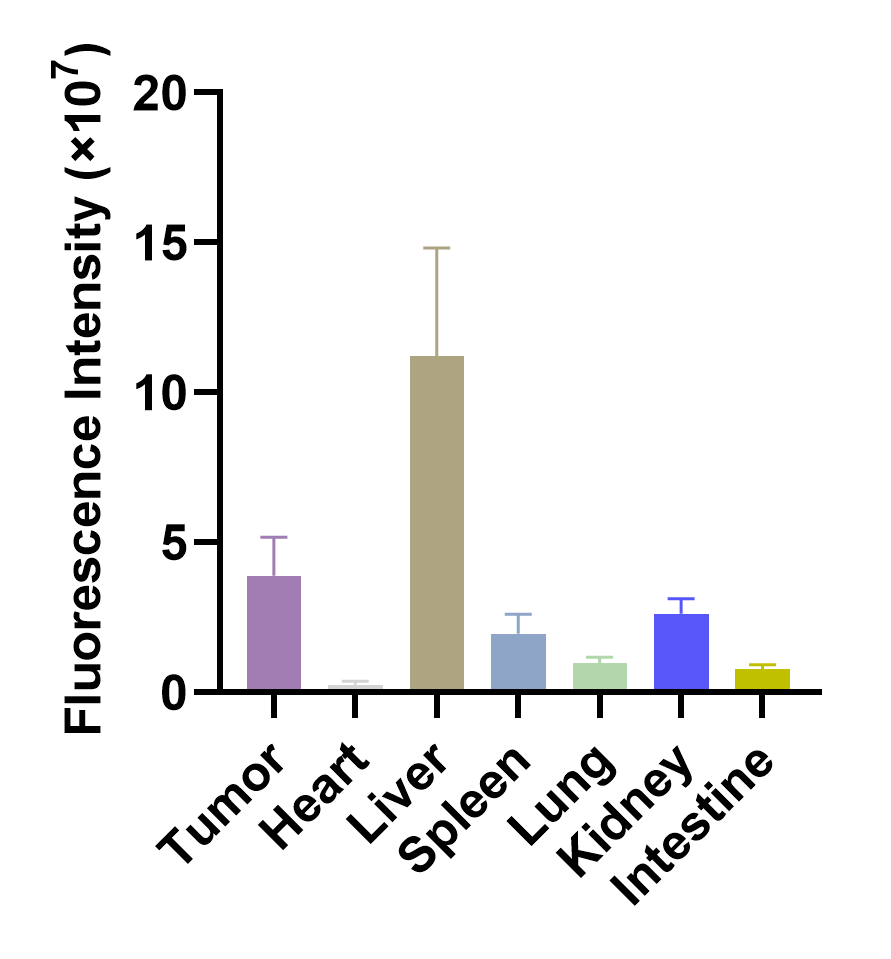


****Fig. S11**** MFI of Pt@PCN-Cu@Cy7.5 in major organs and tumor.


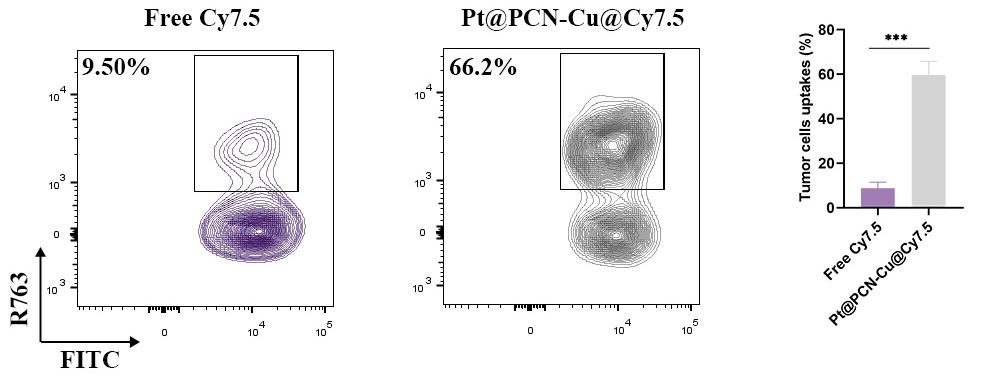


****Fig. S12**** The intracellular accumulation of Pt@PCN-Cu@Cy7.5 in tumor cells was assessed by flow cytometry. Data are presented as mean±SD. Statistical significance was determined using t-test, with ****P*<0.001.

****
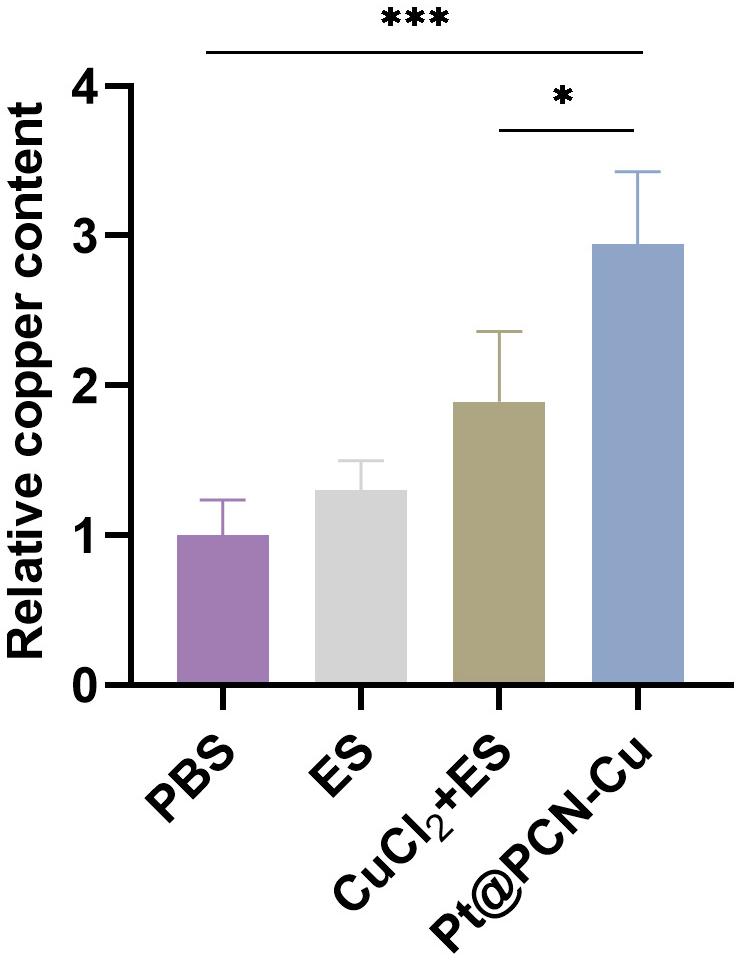
****

****Fig. S13** Copper content in ex vivo tumor tissues detected by ICP-MS.** Data are presented as mean±SD. Statistical significance was determined using one-way ANOVA, with **P*<0.05 and ****P*<0.001.


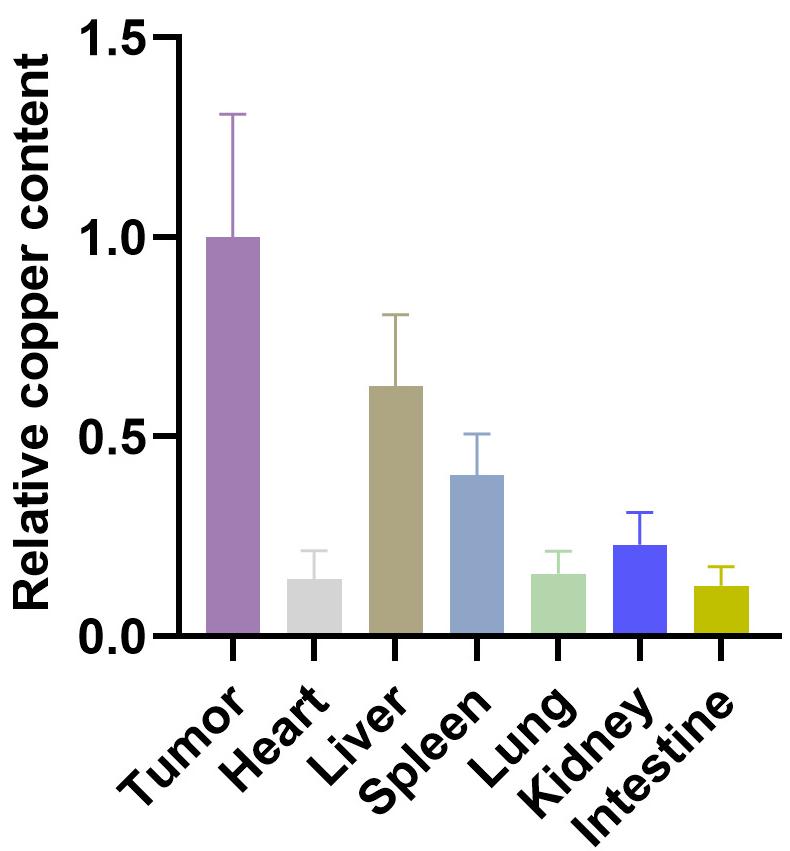


****Fig. S14** The concentration of copper in major organs and tumor after treatments with intravenous injection.**


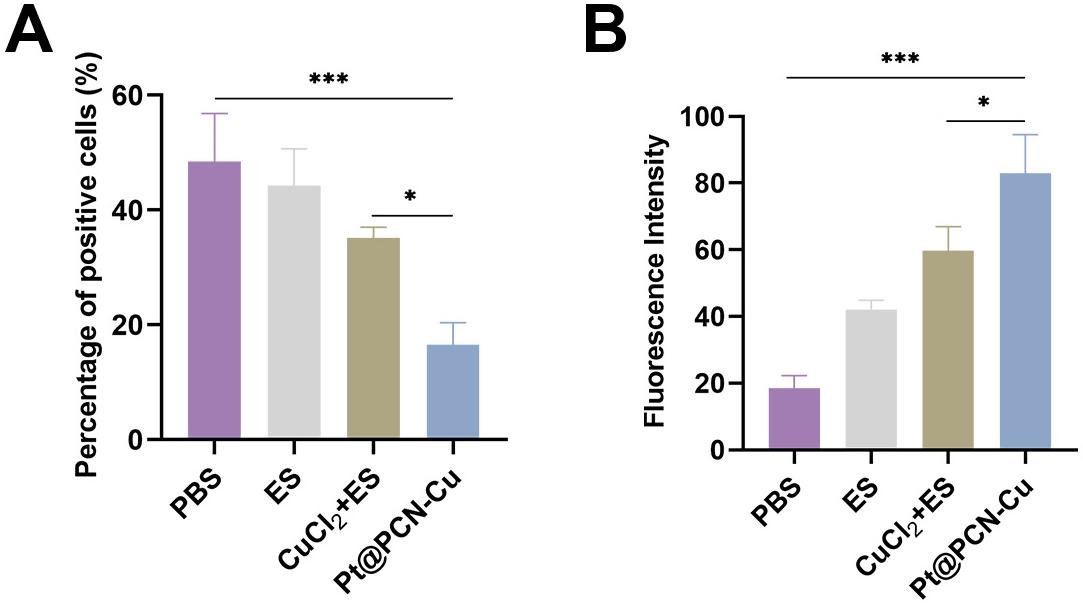


**Fig. S15** (**A**) Quantification of Ki67 expression in tumor tissue from mice given the indicated treatments. (**B**) Quantification of DLAT expression in tumor tissue from mice given the indicated treatments. Data are presented as mean±SD. Statistical significance was determined using one-way ANOVA, with **P*<0.05 and ****P*<0.001.


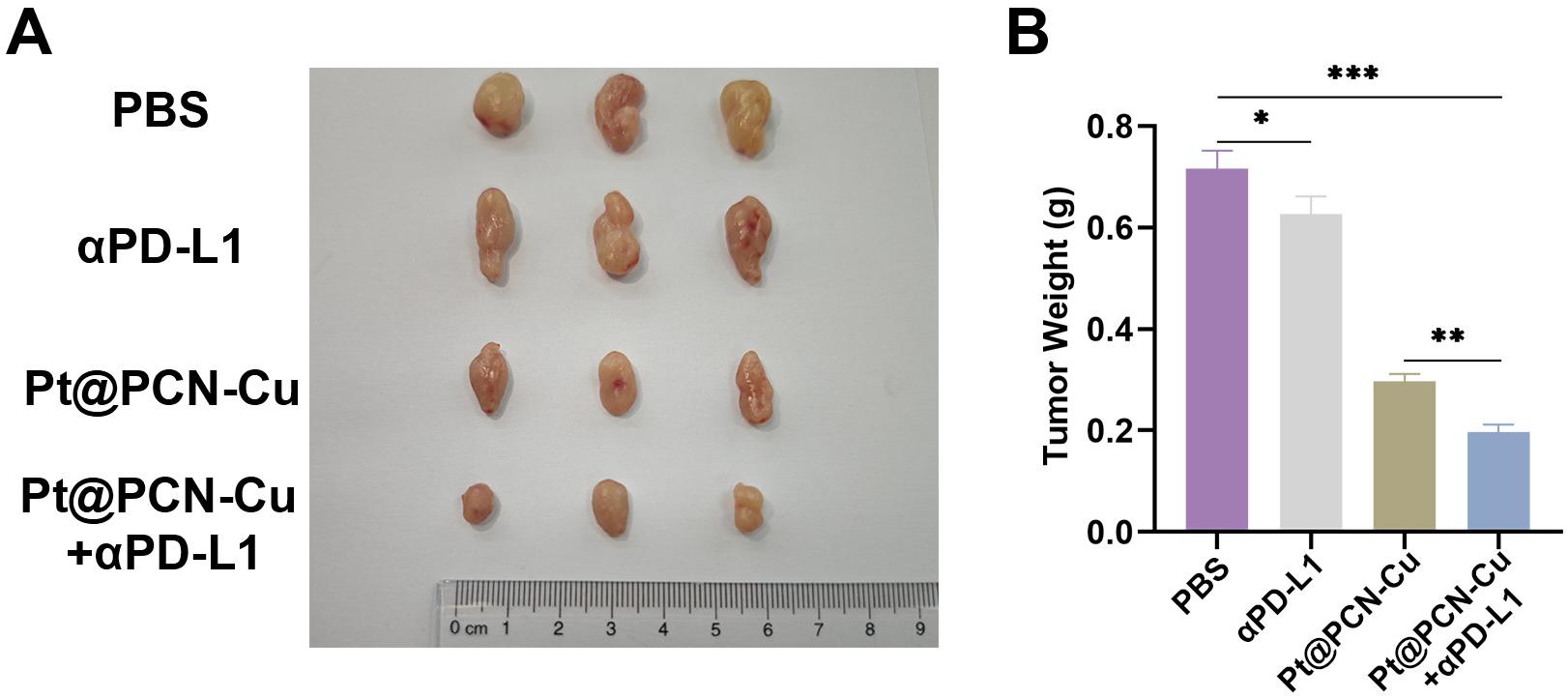


****Fig. S16**** (**A)** Representative photographs of tumors excised from different treatment groups. (**B)** Ex vivo tumor weights measured at the end of the study. Data are presented as mean±SD. Statistical significance was determined using one-way ANOVA, with **P*<0.05, ***P*<0.01 and ****P*<0.001.


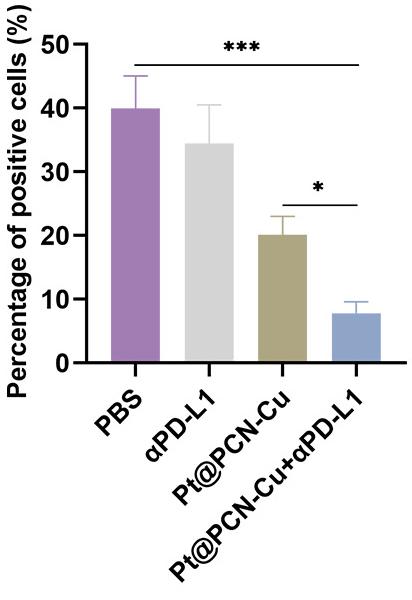


**Fig. S17** Quantification of Ki67 expression in tumor tissue from mice given the indicated treatments. Data are presented as mean±SD. Statistical significance was determined using one-way ANOVA, with **P*<0.05 and ****P*<0.001.


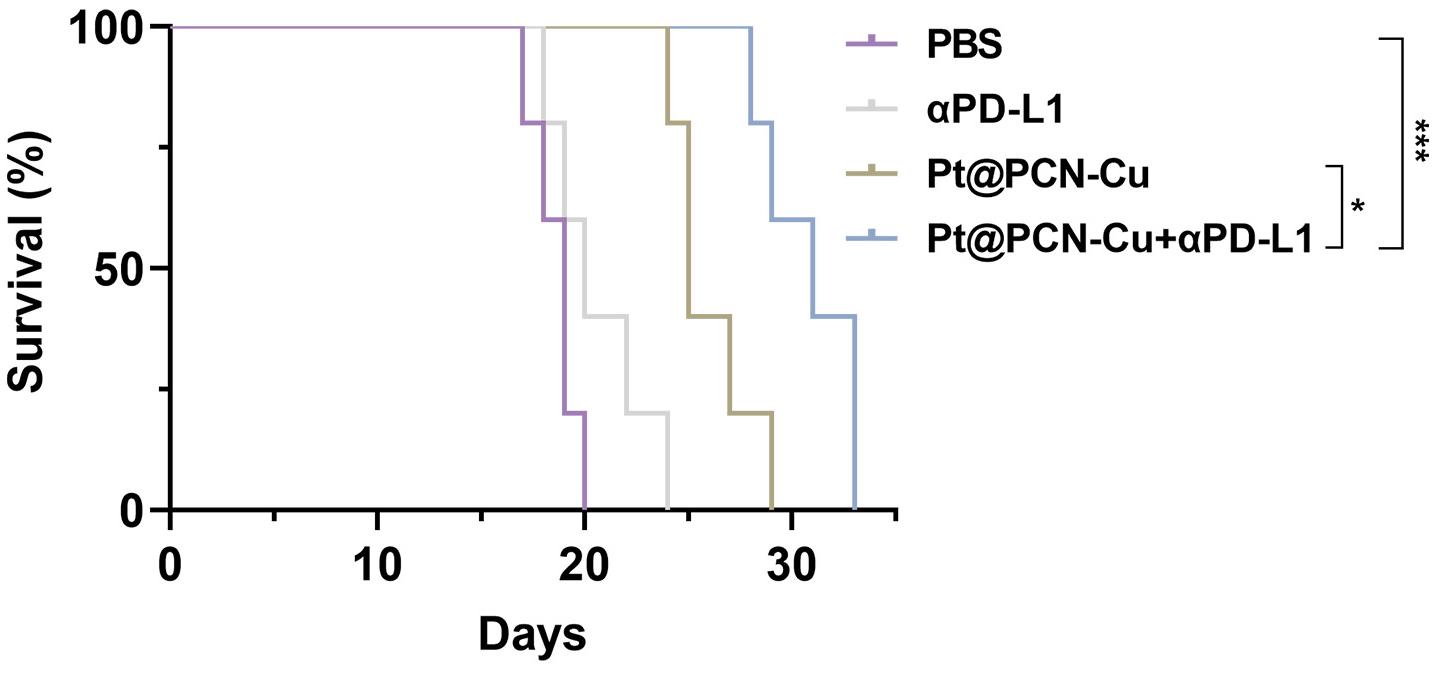


****Fig. S18**** Survival curves of tumor-bearing C57BL/6 mice following different treatments.


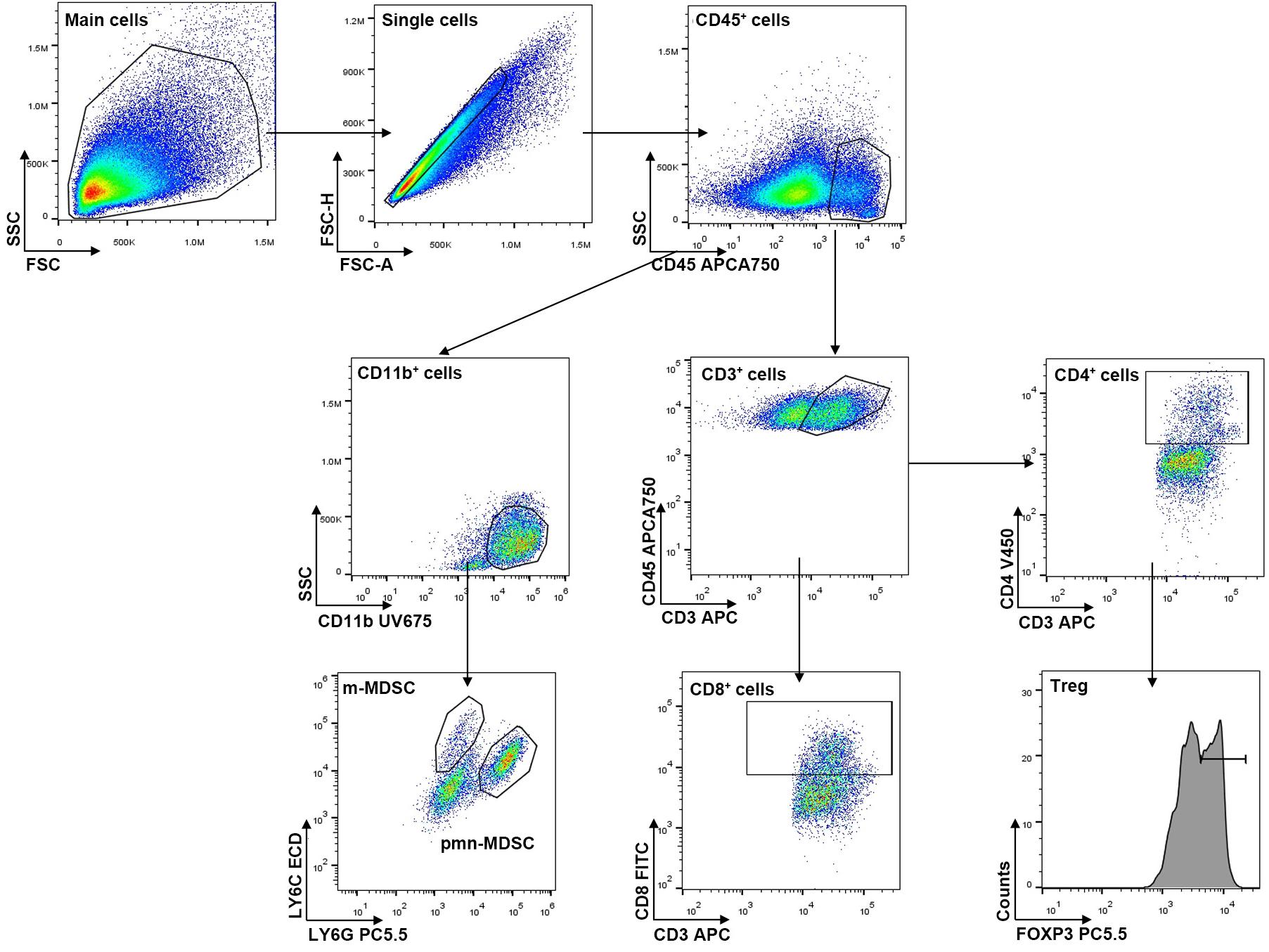


****Fig. S19**** Gating strategies used for flow cytometric analysis.

****
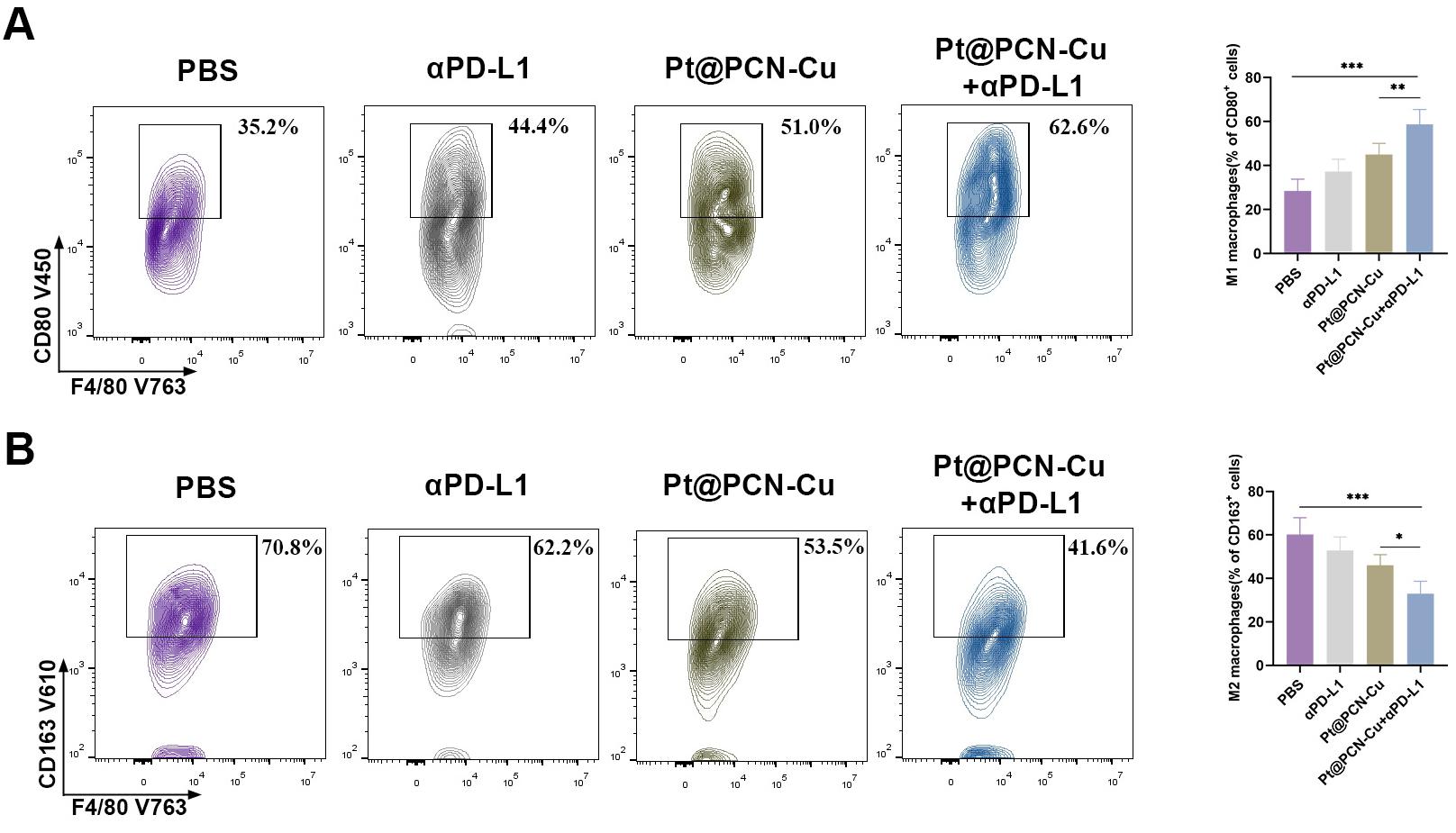
****

****Fig. S20**** (**A**) Quantitative analysis of M1 macrophages within tumors with various treatments. (**B**) Quantitative analysis of M2 macrophages within tumors with various treatments. Data are presented as mean±SD. Statistical significance was determined using one-way ANOVA, with **P*<0.05, ***P*<0.01 and ****P*<0.001.


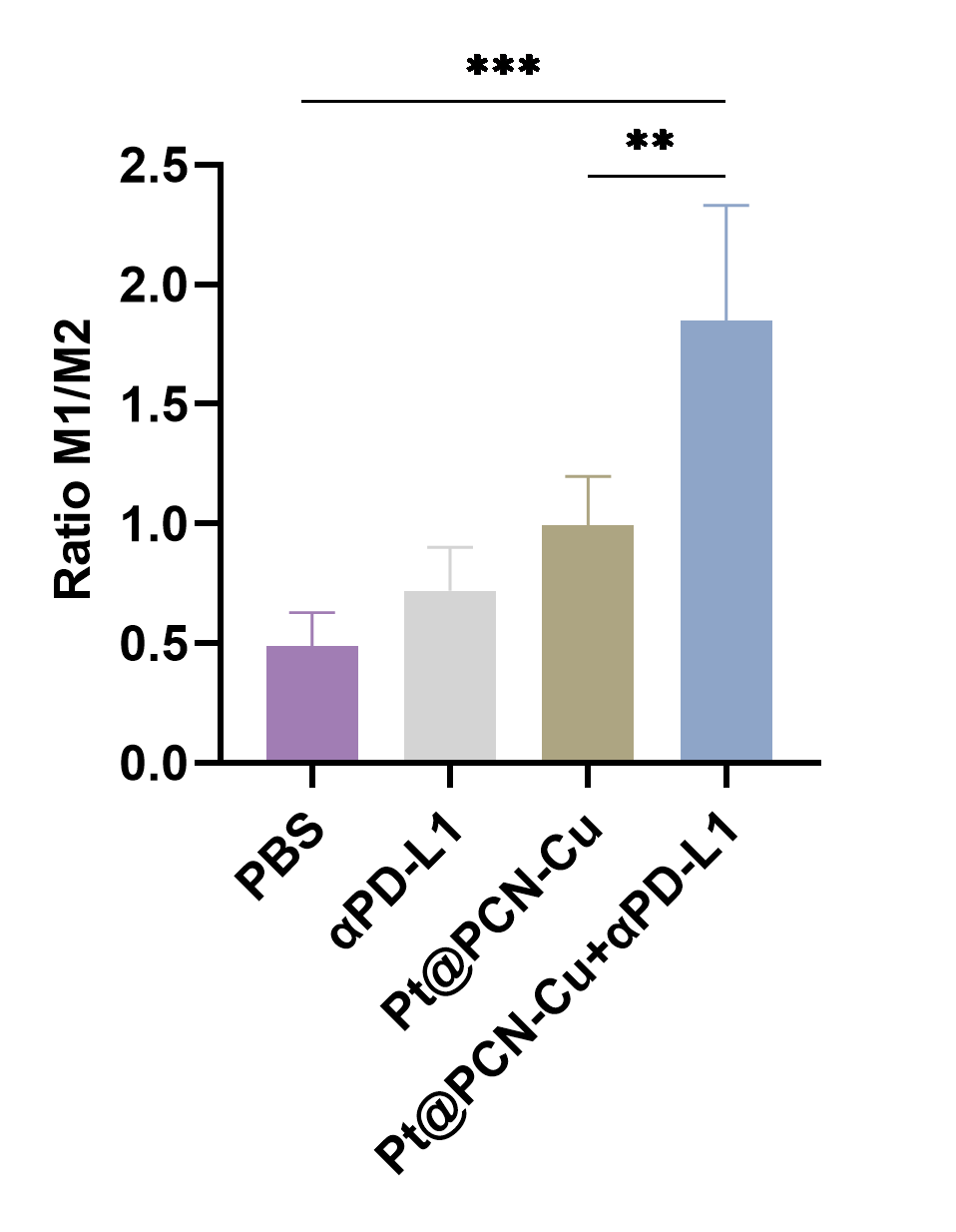


****Fig. S21**** Quantitative analysis of the ratios of M1/M2 macrophages in the tumors with various treatments. Data are presented as mean±SD. Statistical significance was determined using one-way ANOVA, with ***P*<0.01 and ****P*<0.001.
